# METTL3-mediated m6A modification of LINC00839 maintains glioma stem cells and radiation resistance by activating Wnt/β-catenin signaling

**DOI:** 10.1038/s41419-023-05933-7

**Published:** 2023-07-12

**Authors:** Jianxing Yin, Fangshu Ding, Zhangchun Cheng, Xin Ge, Yanhui Li, Ailiang Zeng, Junxia Zhang, Wei Yan, Zhumei Shi, Xu Qian, Yongping You, Zhiliang Ding, Jing Ji, Xiefeng Wang

**Affiliations:** 1 The Affiliated Suzhou Hospital of Nanjing Medical University, Suzhou Municipal Hospital, Gusu School, Nanjing Medical University, Suzhou, Jiangsu 215006 China; 2 Institute for Brain Tumors, Jiangsu Key Laboratory of Cancer Biomarkers, Prevention and Treatment, Collaborative Innovation Center for Cancer Personalized Medicine, Nanjing Medical University, Nanjing, Jiangsu 211166 China; 3 Department of Nutrition and Food Hygiene, Center for Global Health, School of Public Health, Nanjing Medical University, Nanjing, Jiangsu 211166 China; 4 Department of Neurosurgery, The First Affiliated Hospital of Nanjing Medical University, Nanjing, Jiangsu 210029 China; 5 Department of Cancer Biology, The University of Texas MD Anderson Cancer Center, Houston, TX USA; 6 Cancer Hospital, Nanjing Medical University Affiliated Cancer Hospital, Jiangsu Institute of Cancer Research, Nanjing, Jiangsu 210009 China

**Keywords:** CNS cancer, Long non-coding RNAs

## Abstract

Long noncoding RNAs (lncRNAs) are involved in glioma initiation and progression. Glioma stem cells (GSCs) are essential for tumor initiation, maintenance, and therapeutic resistance. However, the biological functions and underlying mechanisms of lncRNAs in GSCs remain poorly understood. Here, we identified that LINC00839 was overexpressed in GSCs. A high level of LINC00839 was associated with GBM progression and radiation resistance. METTL3-mediated m6A modification on LINC00839 enhanced its expression in a YTHDF2-dependent manner. Mechanistically, LINC00839 functioned as a scaffold promoting c-Src-mediated phosphorylation of β-catenin, thereby inducing Wnt/β-catenin activation. Combinational use of celecoxib, an inhibitor of Wnt/β-catenin signaling, greatly sensitized GSCs to radiation. Taken together, our results showed that LINC00839, modified by METTL3-mediated m6A, exerts tumor progression and radiation resistance by activating Wnt/β-catenin signaling.

## Introduction

Glioblastoma (GBM) is the most aggressive and prevalent primary brain tumor in adults [1]. Despite of combinational therapy, including maximal surgical resection, radiotherapy, and chemotherapy, the prognosis of GBM patients remains poor [2]. Glioma stem cells (GSCs) perform a pyramidal hierarchy by self-renewing ability, and have been considered the “seeds” of GBM recurrence for their resistance to radiotherapy and chemotherapy [3–5]. Our previous results demonstrated that GSCs promote GBM TMZ resistance by delivering exosomal miR-30b-3p to tumor cells [6]. Studies have also identified some GSC-specific molecular targets, like bone marrow and X-linked (BMX) signal transducer and activator of transcription 3 (STAT3), contributing to radiotherapy resistance [7, 8]. However, overcoming radiotherapy resistance is still a difficult point in the clinical treatment of glioma [9]. Therefore, more molecules need to be discovered for developing novel therapeutics.

Long noncoding RNAs (lncRNAs) are a class of RNAs consisting of more than 200 nucleotides (nt) and posing a negative ability of encoding proteins [10]. LncRNAs are involved in various biological processes of glioma cells, including proliferation, invasion, chemotherapy, and radiotherapy resistance [11–14]. Although dysregulation of lncRNAs has been reported to participate in the biological functions of GSCs [15], more precise mechanisms of lncRNAs in stemness maintenance and radiotherapy resistance are required to be investigated.

In this study, we identified a novel lncRNA, LINC00839 is involved in stemness maintenance and radiotherapy resistance. Mechanistically, METTL3-YTHDF2 mediated m6A modification elevated LINC00839 levels; LINC00839 functions as a scaffold for c-Src phosphorylates β-catenin at Y654, promoting Wnt signaling activation; β-catenin inhibitor treatment sensitizes brain tumor to radiotherapy.

## Materials and methods

### Clinical specimens

A total of 30 primary GBM specimens and 20 recurrent GBM specimens were obtained from the First Affiliated Hospital of Nanjing Medical University. The use of clinical samples was approved by the medical ethics committee of the First Affiliated Hospital of Nanjing Medical University. The written informed consent have been collected from all patients. Detailed patient information is presented in Supplementary Table 1.

### Cell culture and transfection

Two GSCs (MES28 and GSC2907) and two neural stem cell NSC (HNP1 and NESA), gifted from Professor Xiuxing Wang, were maintained in Neurobasal media (Invitrogen) supplemented with B27 (Invitrogen), EGF, and bFGF (20 ng/ml each; R&D Systems). We synthesized full-length complementary cDNAs of human LINC00839 and cloned into the expression vector pcDNA3.1 (Invitrogen). LINC00839 ASO and control ASO were synthesized by Ribobio. Lipofectamine 3000 reagent (Life Technologies) was used for vector, LINC00839, control ASO, and LINC00839 ASO transfection as suggested by the manufacturer’s protocol. After 48 h of transfection, the cells were collected for analysis.

### RNA extraction and quantitative PCR

Total RNA was extracted from GBM specimens and GSCs using Trizol reagent (Invitrogen) as previously described [6]. The purified RNA was reverse transcribed into cDNA using the MonScriptTM RTIII Super Mix with dsDNase (Monad) according to the manufacturer’s instructions. MonAmp^TM^ Fast SYBR Green qPCR Mix (Monad) and an LC96 Real-Time PCR Detection System (Roche) were used for qRT-PCR analysis. The primers used are listed in Supplementary Table 2.

### Protein extraction and western blotting

Total protein was extracted from GBM specimens and GSCs using RIPA cell lysis buffer (Invitrogen). The FractionPREP Cell Fractionation kit (BioVision) was used for subcellular fractions extraction as previously described [16]. Prepared proteins were separated on SDS- polyacrylamide gel and western blotting assays were performed. Antibodies against LP0 (ab192866, Abcam); Olig2 (65915, CST); SOX2 (3579, CST); MYC (18583, CST); γ-H2AX (9718, CST); WTAP (41934, CST); METTL3 (86132, CST); YTHDF2 (71283, CST); YTHDF1 (43123, CST); YTHDF3 (24206, CST); YTHDC1 (81504, CST); YTHDC2 (46324, CST); EIF3 (3411, CST); hnRNPA2B1 (9304, CST); β-catenin (8480, CST); Flag (SAB4301135, Sigma); c-Src (2109, CST); β-catenin pY654 (ab59430, Abcam); β-catenin pY333 (ab119363, Abcam); E-cadherin (14472, CST); α-Tubulin (3873, CST) were used for western blotting assays.

### Neurosphere formation assay

In vitro limiting dilution was used to measure the Neurosphere formation ability of GSCs as previously described [5]. Briefly, decreasing numbers of GSCs (100, 50, 25, 10, and 2) per well were seeded into a 96-well plate. The presence and number of neurospheres in each well were recorded seven days after plating. Extreme limiting dilution analysis was performed using the ELDA (http://bioinf.wehi.edu.au/software/elda) software.

### Fluorescence in situ hydration (FISH) and immunofluorescence (IF) assay

The co-localization of LINC00839 and YTHDF2 or β-catenin was detected by FISH and IF double staining. Alexa Fluor 555-labeled LINC00839 FISH probe was synthesized by RiboBio. YTHDF2 (71283, CST) and β-catenin (8480, CST) antibodies were used for IF. Images were collected using the fluorescence microscope (Olympus FV1000).

### Clonogenic survival assay

GSCs were radiated at 0, 2, 4, 6, 8, or 10 Gy and seeded into 6-well plates at 500 cells per well in 4 ml. Two weeks later, the formed colonies were fixed with 4% formaldehyde and stained with 0.5% crystal violet. Colony numbers were counted. And the ratio of the plating efficiency of the radiated GSCs to that of control cells was calculated.

### RNA pull-down assay

LINC00839 RNA was transcribed using Ribo™ RNA max-T7 Transcription Kit (RIBOBIO) and Pierce RNA 3’ End desthiobiotinylation Kit (Thermo) was used to label LINC00839. RNA pull-down assays were performed using Pierce^TM^ Magnetic RNA-Protein Pull-Down Kit (Thermo) following the manufacturer’s instructions. Magnetic streptavidin-coated beads were used to extract LINC00839-associated proteins for western blotting assays.

### RNA immunoprecipitation (RIP) assay

RIP assays were performed using the Magna RIP^TM^ Kit (Millipore) according to the manufacturer’s instructions. Briefly, cell lysis was incubated with indicated antibodies-coated magnet beads at 4 °C for 24 h. The protein-associated RNAs were eluted and purified for further qRT-PCR analysis.

### MeRIP assay

MeRIP assays were performed using the Magna MeRIP^TM^ m6A Kit (Millipore) according to the manufacturer’s instructions. Briefly, protein A/G magnetic beads were coated with anti-m6A antibody at 4 °C for 24 h. GSCs RNA was fragmented to 94 °C for 5 min, and incubated with coated beads at 4 °C for 2 h mixture with RNase inhibitor. m6A RNA was eluted and purified for further qRT-PCR analysis.

### Immunoprecipitation (IP) assay

GSCs were lysed using the Pierce™ IP Lysis Buffer (Thermo). Protein A/G magnetic beads were coated with β-catenin antibody, and incubated with cell lysis at 4 °C for 24 h. Proteins were eluted for further western blotting assays.

### In vitro phosphorylation assay

In vitro kinase assay was performed as previously described [17]. Active GST tagged β-catenin (SRP5172) and c-Src (S1076) were purchased from Sigma. Briefly, 5 μg FL or TL LINC00839 RNA and 200 ng active β-catenin were incubated with or without 200 ng c-Src in 50 μl NETN buffer containing 500 μM ATP for 1 h at 30 °C. Reaction results were analyzed by immunoblotting with phosphor-specific antibodies.

### Animal experiments

The animal studies were approved by the Institutional Review Board of Nanjing Medical University (Nanjing, China). All mice were age and sex-matched and then randomized into the different groups. The investigators were not blinded to group allocation during experiments. Briefly, MES28 cells were intracranially injected into athymic nude mice (6-week-old female) as previously described [18]. Mice received 4 Gy of IR on 8, 15, and 22 days after injection using the X-Ray Irradiation System (Faxitron MultiRad 225). For celecoxib treatment, mice were orally administered with or without 90 mg kg^−1^ celecoxib daily. On day 28, after injection, mice were euthanized. And mice brains were harvested, fixed in 4% formaldehyde, embedded in paraffin, and cut into 4 μm sections. Hematoxylin-eosin (HE) staining was performed on sections for histological analysis. Parallel survival experiments were performed to observe the mice until the development of neurological signs.

### Statistical analyses

The statistical analyses were performed using GraphPad Prism. The two-tailed unpaired Student’s t-test or one-way ANOVA with Dunnett’s posttest, and log-rank test were employed. All data represent the mean ± standard deviation (SD) of three independent experiments unless specifically indicated. *P* < 0.05 was considered statistically significant.

## Results

### LINC00839 is upregulated in GSCs

**Fig. 1:**
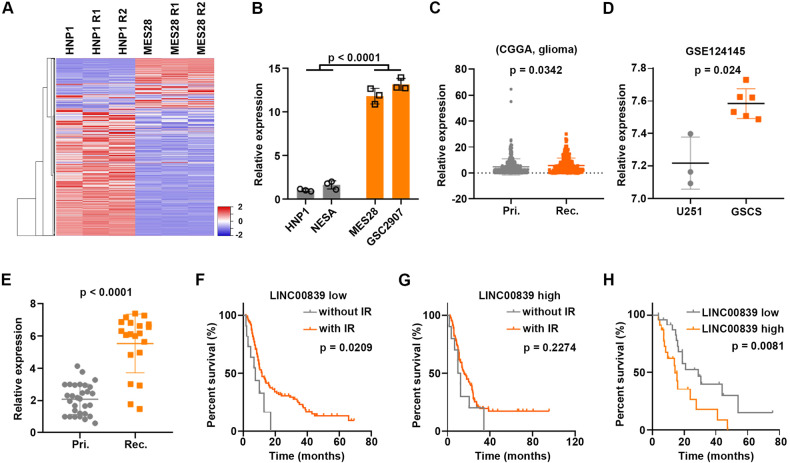
LINC00839 is upregulated in GSCs. **A** Hierarchical clustering of differentially lncRNAs expression in HNP1 and MES28 cells. **B** LINC00839 expression in NSCs and GSCs was detected by qRT-PCR. **C** LINC00839 expression in primary (Pri.) and recurrent (Rec.) glioma samples was measured using the CGGA dataset. **D** LINC00839 expression in U251 cells and GSCs was measured using the GSE124145 dataset. **E** LINC00839 expression in primary (Pri.) and recurrent (Rec.) GBM samples was detected by qRT-PCR. **F**, **G** Glioma samples were divided into two groups: LINC00839 low and high, separated by the median of LINC00839 expression. The correlation between LINC00839 expression and overall survival in glioma patients with or without IR treatment was assessed by Kaplan–Meier survival analysis. **H** The correlation between LINC00839 expression and overall survival in GBM patients was assessed by Kaplan–Meier survival analysis.

To identify the potential oncogenic lncRNAs in GSCs contributing to the malignant phenotype of GSCs, HNP1, and MES28 were collected for RNA-seq. Heatmaps showed a clear distinction between HNP1 and MES28 (Fig. 1A). Top five lncRNAs (LINC00839, PAX8-AS1, MIR4458HG, LINC02732, and ELFN1-AS1) were validated in HNP1, NESA, MES28, and GSC2907. Notably, LINC00839 was mostly increased (about 12-fold) in GSCs than NSCs (Fig. 1B). The other four lncRNAs were increased about 1.5–5-fold in GSCs than NSCs (Supplementary Fig. S1A). Therefore, we focused on LINC00839 in this study.

*LINC00839* is located in 10q11.21 (Supplementary Fig. S1B). The results of 5′ and 3′ rapid amplification of the cDNA ends (RACE) and quantitative real-time PCR (qPCR) revealed that the 2322-nt LINC00839 is the predominant and greatly stable transcript in GSCs (Supplementary Fig. S1C–E). Coding potential analysis result by LNCipedia [19] indicated that LINC00839 is possibly noncoding (Supplementary Fig. S1F). In vitro transcription and translation assay showed that neither the sense nor the antisense transcript of LINC00839 could encode protein (Supplementary Fig. S1G). In addition, the RNA pull-down assay indicated no interaction between LINC00839 and ribosomal protein LP0 (Supplementary Fig. S1H). These results confirmed that LINC00839 is a bona fide noncoding RNA.

Next, CGGA dataset analysis results indicated that LINC00839 was significantly increased in recurrent glioma tissues (Fig. 1C). Meanwhile, GSE124145 dataset analysis results showed that LINC00839 was increased in GSCs than U251 cells (Fig. 1D). qRT-PCR results also showed that LINC00839 was significantly increased in recurrent GBM samples (Fig. 1E). Glioma patients with lower levels of LINC00839 exhibited better response to IR (Fig. 1F, G). Importantly, high LINC00839 level is remarkably associated with poor prognosis of GBM patients (Fig. 1H). These results confirm the involvement of LINC00839 in GSCs maintenance, glioma growth, IR resistance, and brain glioma recurrence.

### LINC00839 facilitates GSC maintenance and radiation resistance in vitro and in vivo

**Fig. 2:**
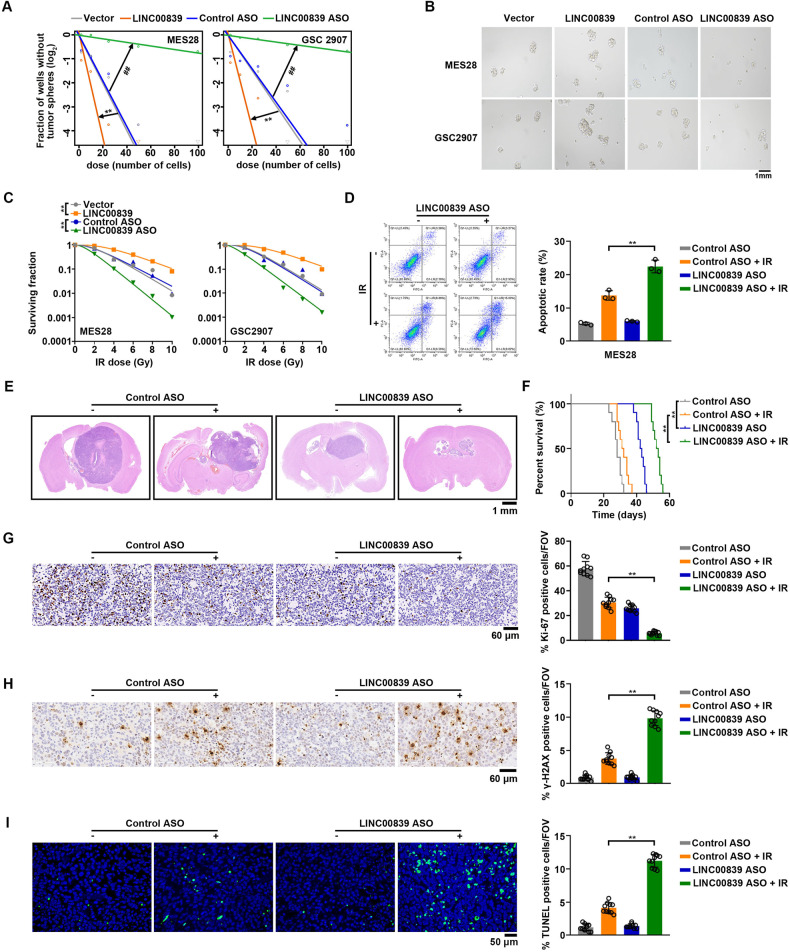
LINC00839 facilitates GSC maintenance and radiation resistance in vitro and in vivo. **A** GSCs were transfected with Vector, LINC00839, control ASO, or LINC00839 ASO separately. Effects on self-renewal were assessed by in vitro extreme limiting dilution assays (ELDA) for sphere formation.* refers to compare between the Vector group and the LINC00839 group; # refers to compare between the control ASO group and LINC00839 ASO group. **p < 0.001, ##*p* < 0.001. **B** GSCs were transfected with Vector, LINC00839, control ASO, or LINC00839 ASO separately. Representative images of spheres were photographed. **C** GSCs were transfected with Vector, LINC00839, control ASO, or LINC00839 ASO separately. Cells were treated with or without IR (0–10 Gy) and seeded in 10 cm dishes. Colonies were counted after 2 weeks, and the surviving fraction was calculated as the ratio of the plating efficiency of the treated cells to that of control cells. **p < 0.001. **D** GSCs were transfected with control ASO or LINC00839 ASO. The apoptotic rates were measured by flow cytometry. **p < 0.001. **E** Representative images of hematoxylin and eosin stained cross-sections of tumor-bearing brains harvested after transplantation of transfected GSCs with or without IR treatment. **F** Kaplan–Meier survival curves of immunocompromised mice-bearing intracranial injected GSCs. **p < 0.001. **G**, **H** Representative IHC images and the quantification of Ki-67 (**G**) and γ-H2AX (**H**) were shown. Scale bars, 60 μm. **p < 0.001. **I** Representative TUNEL images and the quantification were shown. Scale bars, 60 μm. **p < 0.001.

To elucidate the functional role of LINC00839 in radiation resistance, we stably overexpressed or knocked down LINC00839 in GSCs (Supplementary Fig. S2A). The results of extreme limiting dilution assays (ELDA) suggested that LINC00839 overexpression markedly enhanced GSC frequency and self-renewal (Fig. 2A). LINC00839 overexpression also promoted sphere formation in GSCs (Fig. 2B). At the same time, stemness markers (Olig2, Sox2, and MYC) expression increased after overexpressing LINC00839 (Supplementary Fig. S2B). LINC00839 knockdown exhibited the opposite effect on stemness maintenance (Fig. 2A, B and Supplementary Fig. S2C).

Clonogenic survival analysis were performed to evaluate the functional role of LINC00839 in regulating the IR resistance of GSCs. The results indicated that LINC00839 overexpression resulted in decreased sensitivity of GSCs to IR (Fig. 2C). Consistently, LINC00839 overexpression induced decreased apoptotic rate and shortened γ-H2AX level upon IR treatment (Supplementary Fig. S2D, F, G). On the contrary, LINC00839 knockdown led to sensitivity to IR reflected in decreased clonogenic survival rate (Fig. 2C), increased apoptotic rate (Fig. 2D and Supplementary Fig. S2E), and sustained γ-H2AX level upon IR treatment (Supplementary Fig. S2H).

Mice-bearing intracranial xenografts derived from LINC00839 knockdown MES28 cells showed a drastic decrease in tumor volume and prolonged survival time (Fig. 2E, F). Ki-67 levels were dramatically decreased caused by LINC00839 knockdown (Fig. 2G). Additionally, tumors from LINC00839 knockdown GSCs exhibited a better response to IR treatment (Fig. 2E, F), accompanied by increased γ-H2AX and apoptosis (Fig. 2H, I).

In summary, these results showed that loss of LINC00839 in GSCs resulted in impaired stemness and increased radiation sensitivity to IR.

### METTL3-mediated m6A modification enhances LINC00839 expression

**Fig. 3:**
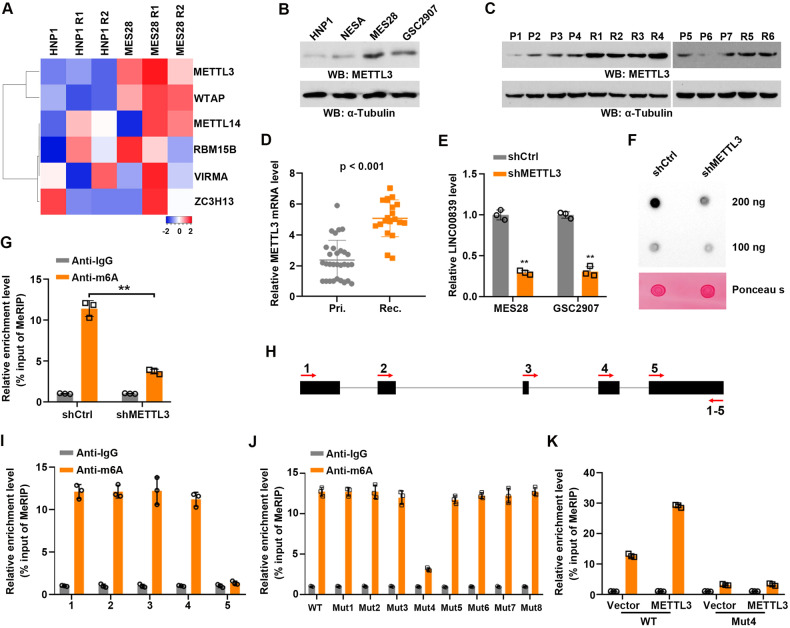
METTL3-mediated m6A modification enhanced LINC00839 expression. **A** Hierarchical clustering of differentially “writers” expression in HNP1 and MES28 cells. **B** METTL3 expression in NSCs and GSCs was detected by western blot. **C** METTL3 expression in primary (P) and recurrent (R) GBM samples was detected by western blot. **D** METTL3 expression in primary (Pri.) and recurrent (Rec.) GBM samples was detected by qPCR. **E** GSCs were treated with control or METTL3 shRNA. LINC00839 expression was detected by qRT-PCR. **p < 0.001. **F** Total RNA m6A contents in LINC00839-knockdown GSCs was quantified by dot blot assays. **G** m6A enrichment in LINC00839 transcripts in control and METTL3-silencing cells using MeRIP-qPCR. **H** Primer-walking strategy. Five paired primers were designed according to the gene/exon–intron length. **I** MeRIP-qPCR assays using walking primers were performed to analyze the m6A-modification region. **J** MeRIP-qPCR assays to analyze the m6A-modification levels of LINC00839 in MES28 transfected with LINC00839 wildtype and its mutants expression. **K** MeRIP-qPCR assays to analyze the m6A-modification levels of LINC00839 in control or METTL3-depleted MES28 transfected with LINC00839 wildtype and its mutants expression.

N6-methyladenosine (m6A) modification has been considered as the most prevalent internal RNA modification in tumor cells [20]. Previous studies have revealed the important roles of m6A modification in the regulation of RNA degradation, stability, and splicing [11, 21, 22]. RNA-seq results indicated that METTL3 and WTAP were elevated in GSCs compared with NSCs, while there’s no significant difference in other writers between GSCs and NSCs (Fig. 3A). Furthermore, the mRNA and protein levels of METTL3 and WTAP in GSCs were verified, and the results showed that METTL3, instead of WTAP, was remarkably increased in GSCs compared with those in NSCs (Fig. 3B and Supplementary Fig. S3A–C). In addition, METTL3 levels were higher in recurrent GBM tissues than in primary samples (Fig. 3C, D). These results suggested that METTL3 is involved in stemness maintenance and IR resistance of GSCs.

Next, we tend to explore the post-transcriptional regulation of METTL3 on LINC00839. METTL3 was depleted or overexpressed in GSCs (Supplementary Fig. S3D, E). METTL3 knockdown significantly suppressed LINC00839 levels in GSCs (Fig. 3E); while METTL3 overexpression elevated LINC00839 levels in GSCs (Supplementary Fig. S3F). These results suggested that METTL3 positively regulates LINC00839.

To explore the potential mechanism of METTL3 regulating LINC00839, we first performed m6A quantification assays and found that METTL3 silencing induced a great decrease in global m6A levels in GSCs (Supplementary Fig. S3G), which was confirmed by the m6A dot blot assays (Fig. 3F). Methylated RNA immunoprecipitation (MeRIP) assay showed that METTL3 knockdown induced a great decrease of m6A modification on LINC00839 (Fig. 3G). Methylated RNA immunoprecipitation (MeRIP) assay followed by qRT-PCR using primer-walking indicated that METTL3 mediated m6A modification located in Exon 4 of LINC00839 (Fig. 3H, I). These results indicated METTL3 promotes LINC00839 expression by m6A modification.

Using the online tool SRAMP [23], we found 7 sites (A679, 692, 840, 1075, 1119, 1130, and 1242) were predicted to be m6A modification sites, and they were distributed in exon 3, 4, and 5 of the LINC00839 (Supplementary Fig. S3H). To determine the accurate m6A modification sites on LINC00839, we constructed a wildtype and 8 mutant LINC00839 plasmids, in which the adenine residues in predicted m6A motifs in LINC00839 were substituted by guanine (A–G mut) (Supplementary Fig. S3I). Then, the PiggyBac transposon system was used to transfect plasmids into LINC00839 knockdown GSCs (Supplementary Fig. S3J). MeRIP-qPCR showed that Mut 4 led to apparently decreased m6A levels on LINC00839, suggesting that site 4 was responsible for METTL3-mediated m6A modification (Fig. 3J). In addition, the site 4 mutation abrogated the promoting effect of METTL3 on m6A modification on LINC00839 (Fig. 3K).

Collectively, our findings revealed that METTL3-mediated m6A modification enhanced LINC00839 expression in GSCs.

### YTHDF2 stabilizes LINC00839

**Fig. 4:**
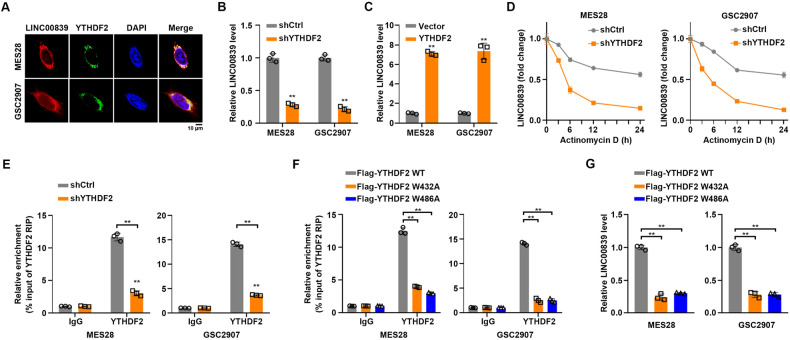
YTHDF2 stabilizes LINC00839. **A** FISH and IF double staining in GSCs shows the co-localization of LINC00839 (Cy3; Red) and YTHDF2 (Green); Nuclei are stained blue (DAPI). Scale bar: 10 μm. **B** LINC00839 expression in GSCs transfected with control or YTHDF2 shRNA was detected by qRT-PCR. **p < 0.001. **C** LINC00839 expression in GSCs transfected with vector or YTHDF2 was detected by qRT-PCR. **p < 0.001. **D** LINC00839 RNA stability in control and YTHDF2-silenced cells. qRT-PCR of LINC00839 at the indicated time points after treatment with actinomycin D (10 μg/mL). **E** YTHDF2 was immunoprecipitated and RIP-qPCR was used to assess the association of LINC00839 with YTHDF2. **p < 0.001. **F** Enrichment of LINC00839 in the immunoprecipitated RNA fraction of GSCs following overexpression of either wildtype (WT) YTHDF2 or m6A-binding mutant YTHDF2 (W432A and W486A) in GSCs. **p < 0.001. **G** LINC00839 expression in GSCs expressing either WT or mutant YTHDF2 was detected by qRT-PCR. **p < 0.001.

To identify the m6A reader that accounts for the m6A modification on LINC00839, LINC00839-based RNA pull-down assays were performed in MES28 and GSC2907. m6A readers in the pull-down products of LINC00839 were examined, and the results showed the direct interaction between LINC00839 and YTHDF2 instead of other readers (Supplementary Fig. S4A). YTHDF2 belongs to the YTH domain family, and has been validated as an important m6A “reader” [24]. YTHDF2 has been demonstrated to regulate the degradation of target mRNAs or lncRNAs [18, 25]. RNA-Protein Interaction Prediction (RPISeq) software predicted strong interaction probabilities, with a score >0.5 (Fig. S4B). Fluorescent in situ hybridization (FISH) accompanied with IF further confirmed the co-localization of LINC00839 and YTHDF2 in the cytoplasm (Fig. 4A). Consistent with previous results [18], expression of YTHDF2 was upregulated in GSCs and recurrent gliomas (Supplementary Fig. S4C, D). YTHDF2 inhibition significantly decreased LINC00839 levels in GSCs (Supplementary Fig. S4E and Fig. 4B); while YTHDF2 overexpression increased LINC00839 levels in GSCs (Supplementary Fig. S4F and Fig. 4C). To determine whether YTHDF2 regulates the stability of LINC00839, we treated GSCs with actinomycin D to arrest transcription. The decay rate of LINC00839 was higher upon YTHDF2 depletion (Fig. 4D), suggesting that YTHDF2 is critical for the stabilization of LINC00839.

Next, we tend to further establish the role of YTHDF2 in METTL3-mediated LINC00839 upregulation. We performed RNA immunoprecipitation (RIP) assays and found that LINC00839 was markedly enriched using anti-YTHDF2 antibodies, which was undermined when YTHDF2 silenced (Fig. 4E). Next, we depleted and reconstituted m6A-binding mutant YTHDF2 constructs (W432A and W486A) in GSCs (Supplementary Fig. S4G). RIP assay results showed that W432A and W486A mutation reduced the binding of YTHDF2 to LINC00839 (Fig. 4F). qRT-PCR results indicated that W432A and W486A mutation significantly decreased LINC00839 levels in GSCs (Fig. 4G).

Taken together, YTHDF2 is essential for LINC00839 stability and expression in GSCs.

### LINC00839 interacts with β-catenin in GSCs

**Fig. 5:**
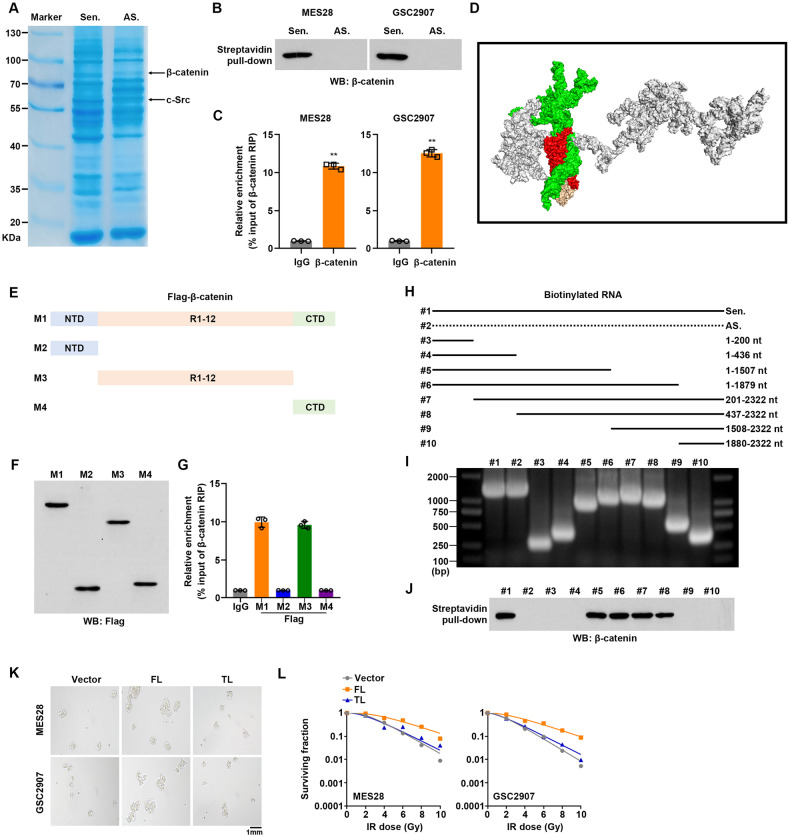
LINC00839 interacts with β-catenin in GSCs. **A** Coomassie blue staining of the LINC00839–protein complex by incubation of biotinylated-LINC00839 with protein extracts from MES28. **B** The enrichment of β-catenin in pull-down products of LINC00839 was measured by western blot. **C** RNA immunoprecipitation with an anti-β-catenin antibody was used to assess whether β-catenin binding to LINC00839 in GSCs. **D** Molecular docking diagram of LINC00839 and β-catenin. Green: 445–1503 nt fragment of LINC00839; Red: 127–376 residues of β-catenin. **E**–**G** Deletion mapping for the domains of β-catenin that bind to LINC00839. RIP analysis for LINC00839 enrichment in cells transiently transfected with FLAG-tagged full-length or truncated constructs. **H**–**J** Immunoblotting of β-catenin in pull-down samples by full-length biotinylated-LINC00839 (#1), biotinylated-LINC00839 antisense (#2), or truncated biotinylated-LINC00839 RNA motifs (#3–10). **K** GSCs were transfected with FL or TL LINC00839. Representative images of spheres were photographed. Scale bars, 1 mm. **L** GSCs were transfected with FL or TL LINC00839. Cells were treated with or without IR (0–10 Gy) and seeded in 10 cm dishes. Colonies were counted after 2 weeks, and the surviving fraction was calculated as the ratio of the plating efficiency of the treated cells to that of control cells.

To explore the molecular mechanism underlying the oncogenic activity of LINC00839 in GSCs radiation resistance, we performed LC-MS to identify the proteins associated with LINC01138 in GSCs. The results showed that sense but not antisense LINC00839, was specifically associated with β-catenin (Fig. 5A and Supplementary Table 3). This interaction was confirmed in MES28 and GSC2907 by streptavidin-LINC00839-based RNA pull-down (Fig. 5B). We observed a co-distribution of β-catenin and LINC00839 in GSCs by IF (Supplementary Fig. S5A). The interaction of LINC00839 and β-catenin was confirmed by RIP assays (Fig. 5C). HDOCK tool was used to predict the interaction structure between LINC00839 and β-catenin, and the results indicated that 127–376 residues of β-catenin could bind with 445–1503 nt fragment of LINC00839 (Fig. 5D and Supplementary Table 4). Next, we constructed full-length and truncated β-catenin based on its secondary structure (Fig. 5E, F). RIP assay results showed that LINC00839 directly binds to the Armadillo repeats domain, instead of NTD and CTD domains (Fig. 5G). Furthermore, a series of LINC00839 deletion was constructed according to its secondary structure (Fig. 5H, I). RNA pull-down results indicated that the 437–1507-nt fragment mediates the capability to bind to β-catenin as efficiently as the full-length LINC00839 (Fig. 5J). To emphasize the critical role of the 437–1507-nt fragment of LINC00839 in mediating radiation resistance, we overexpressed full-length (FL) or 437–1507-nt fragment truncated-length (TL) LINC00839 in GSCs. 437–1507-nt fragment deletion significantly impaired the improved stemness of GSCs induced by FL LINC00839 transfection, as reflected by tumor sphere formation and stemness markers detection (Fig. 5K and Supplementary Fig. S5C, D). Moreover, clonogenic survival analysis, flow cytometry analysis, and γ-H2AX detection results indicated that 437–1507-nt fragment of LINC00839 is sufficient for maintaining IR resistance in GSCs (Fig. 5L and Supplementary Fig. S5E, F).

Taken together, our results indicate that LINC00839 physically interacts with β-catenin to promote GSC maintenance and radiation resistance.

### LINC00839 promotes c-Src-mediated β-catenin phosphorylation

Next, we tend to characterize the molecular consequences of the interaction of LINC00839 and β-catenin. Firstly, we found that neither of LINC00839 overexpression nor knockdown had significant effects on mRNA and protein levels of β-catenin (Supplementary Fig. S6A–D). Substrate distribution analysis results indicate that LINC00839 knockdown increased the membrane retention of β-catenin (Supplementary Fig. S6E), leading to the suppressed activation of Wnt signaling (Supplementary Fig. S6F). Consistently, METTL3 or YTHDF2 knockdown significantly inhibited activation of Wnt signaling, while LINC00839 could co-transfection could partially reverse this effect (Supplementary Fig. S6G, H). Moreover, FL, not TL LINC00839 overexpression, promoted membrane disassociation of β-catenin (Supplementary Fig. S6I) and activation of Wnt signaling in GSCs (Supplementary Fig. S6J).

**Fig. 6:**
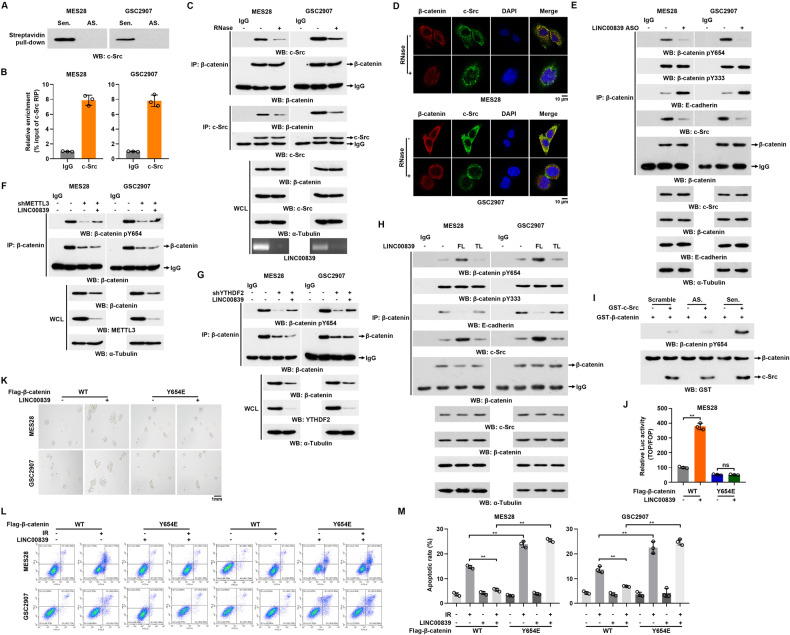
LINC00839 promotes c-Src-mediated β-catenin phosphorylation. **A** The enrichment of c-Src in pull-down products of LINC00839 was measured by western blot. **B** RNA immunoprecipitation with an anti-c-Src antibody was used to assess whether c-Src binding to LINC00839 in GSCs. **C** GSCs were transfected with or without RNase I_f_. Immunoprecipitation with anti-β-catenin and anti-c-Src antibodies were performed. **D** GSCs were transfected with or without RNase I_f_. IF assays were performed to analyze the co-localization of β-catenin (Red) and c-Src (Green). **E** GSCs were transfected with control or LINC00839 ASO. Immunoprecipitation with an anti-β-catenin antibody was performed. **F** Control or METTL3 deleted GSCs were co-transfected with LINC00839. Immunoprecipitation with an anti-β-catenin antibody was performed. **G** Control or YTHDF2 deleted GSCs were co-transfected with LINC00839. Immunoprecipitation with an anti-β-catenin antibody was performed. H. GSCs were transfected with FL or TL LINC00839. Immunoprecipitation with an anti-β-catenin antibody was performed. **I** In vitro β-catenin phosphorylation assay using recombinant β-catenin, c-Src proteins, and in vitro-transcribed RNA transcripts as indicated in NETN buffer with the presence of 500 µM ATP. Immunoblots were used to detect β-catenin phosphorylation level. **J** Luciferase activity (TOP/FOP) in control or LINC00839 overexpressing MES28 co-transfected with WT or Y654E mutation β-catenin. **p < 0.001. **K** Control or LINC00839 overexpressing GSCs were co-transfected with WT or Y654E mutation β-catenin. Representative images of spheres were photographed. Scale bars, 1 mm. **L**, **M** Control or LINC00839 overexpressing GSCs were co-transfected with WT or Y654E mutation β-catenin. The apoptotic rates were measured by flow cytometry. **p < 0.001.

Previous studies have found that posttranslational modifications (PTMs) are essential for nuclear localization [26]. Accidentally, HPLC-MS results showed the interaction between LINC00839 and c-Src in GSCs (Supplementary Table 4). This interaction was confirmed using RNA pull-down and RIP assays (Fig. 6A, B). c-Src is a member of non-receptor tyrosine kinases, and is overactivated in most cancers [27]. Once Wnt3a and Wnt5a bind to the receptors, c-Src is activated. Activated c-Src binds to β-catenin, decreasing E-cadherin binding, promoting nuclear transfer and Wnt signaling activation [28]. Cell lysates were treated with ribonuclease If (RNase If), followed by co-IP and IF assays. The results showed that RNase If treatment abolished c-Src/β-catenin complex formation (Fig. 6C, D), indicating that the interaction between c-Src and β-catenin was dependent on the existence of LINC00839. Therefore, we tend to investigate whether LINC00839 functions as a scaffold contributing to the tight interaction between c-Src and β-catenin.

A co-immunoprecipitation assay demonstrated that LINC00839 knockdown inhibited the phosphorylation of c-Src on β-catenin Y654 instead of Y333, both residues have been identified as phosphorylation sites of c-Src on β-catenin [28] (Fig. 6E). METTL3 or YTHDF2 knockdown decreased Y654 phosphorylation levels of β-catenin, while LINC00839 co-transfection could partially restore the phosphorylation levels (Fig. 6F, G). Meanwhile, LINC00839 knockdown impaired the association between β-catenin and E-cadherin (Fig. 6E). On the contrary, FL, not TL LINC00839 overexpression increased β-catenin Y654, not Y333, phosphorylation levels in GSCs (Fig. 6H). E-cadherin interaction was impaired after FL LINC00839 transfection (Fig. 6H). In vitro phosphorylation assay results confirmed that LINC00839 increased β-catenin Y654 phosphorylation (Fig. 6I). Moreover, we depleted and reconstituted expression of β-catenin WT or Y654E in GSCs (Supplementary Fig. S6K). Consistent results demonstrated that LINC00839-induced Wnt signaling activation was disrupted by β-catenin Y654E (Fig. 6J and Supplementary Fig. S6L). Tumor sphere formation and stemness markers detection results suggested that β-catenin Y654 phosphorylation was essential for LINC00839 promoting GSC maintenance (Fig. 6K and Supplementary Fig. S6M). Consistently, radiation resistance induced by LINC00839 was abrogated by β-catenin Y654E (Fig. 6L, M and Supplementary Fig. S6N).

Taken together, these results suggested that LINC00839 promotes GSC maintenance and radiation resistance through c-Src-mediated β-catenin phosphorylation.

### Blocking LINC00839-Wnt/β-catenin impaired GBM growth and sensitizes GSCs to IR

**Fig. 7:**
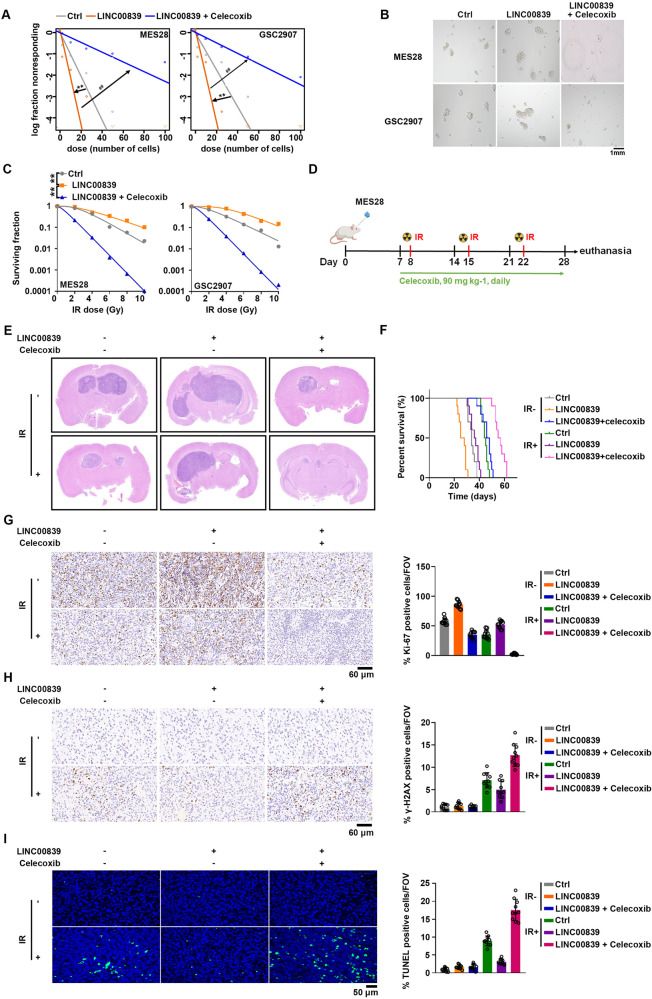
Wnt/β-catenin inhibitor inhibits GBM growth and sensitizes GSCs to IR. **A** Vector or LINC00839 overexpressing GSCs were treated with or without celecoxib. Effects on self-renewal were assessed by in vitro extreme limiting dilution assays (ELDA) for sphere formation. * refers to compare between Vector group and LINC00839 group; # refers to compare between LINC00839 group and LINC00839 + celecoxib group. **p < 0.001, ##*p* < 0.001. **B** Vector or LINC00839 overexpressing GSCs were treated with or without celecoxib. Representative images of spheres were photographed. Scale bars, 1 mm. **C** Vector or LINC00839 overexpressing GSCs were treated with or without celecoxib. Cells were treated with IR (0–10 Gy) and seeded in 10 cm dishes. Colonies were counted after 2 weeks, and the surviving fraction was calculated as the ratio of the plating efficiency of the treated cells to that of control cells. **p < 0.001. **D** Vector or LINC00839 overexpressing MES28 cells were intracranially injected into nude mice (*n* = 10 for each group). Schematic diagram of the treatment of radiation and celecoxib in tumor-bearing mice. **E** Representative images of hematoxylin and eosin stained cross-sections of tumor-bearing brains after radiation and celecoxib treatment. **F** Kaplan–Meier survival curves of immunocompromised mice-bearing intracranial injected GSCs after radiation and celecoxib treatment. **p < 0.001. **G**, **H** Representative IHC images and the quantification of Ki-67 (**G**) and γ-H2AX (**H**) were shown. Scale bars, 60 μm. **p < 0.001. **I** Representative TUNEL images and the quantification were shown. Scale bars, 50 μm. **p < 0.001.

Given that LINC00839 confers GSCs radiotherapy resistance by activating Wnt/β-catenin signaling, we tested whether β-catenin inhibitor could reverse the function of LINC00839. Celecoxib was chosen for further study on account of its ability to transport across the blood–brain-barrier (BBB) and inhibit Wnt signaling activation [29]. In addition, GSCs exhibit a well response to celecoxib treatment (Supplementary Fig. S7A). In consistent with the previous report, celecoxib treatment inhibited Wnt signaling activation in GSCs (Supplementary Fig. S7B). Celecoxib treatment significantly impaired the elevated GSCs self-renewal by LINC00839 overexpression, as measured by ELDA and tumor sphere formation assays (Fig. 7A, B), and stemness markers detection (Supplementary Fig. S7C). Moreover, celecoxib restored the sensitivity of GSCs to radiation treatment as demonstrated by reduced ability of colony formation (Fig. 7C), increased apoptosis rate (Supplementary Fig. S7D), and sustained levels of γ-H2AX (Supplementary Fig. S7E).

We next tested the preclinical effects of celecoxib on sensitizing radiation therapy in vivo (Fig. 7D). The results showed that LINC00839 overexpression promoted tumor growth and radiation resistance; while celecoxib treatment efficiently suppressed tumor growth, whereas celecoxib and irradiation combinational treatment exhibited the strongest tumor inhibition (Fig. 7E). Consistently, tumor-bearing mice treated with celecoxib and irradiation combination longer survival extension when compared to other groups (Fig. 7F). The immunohistochemical results of Ki-67 and γ-H2AX demonstrated that the combination of celecoxib treatment with radiation resulted in stronger growth inhibition and apoptosis of tumor cells in GSCs derived xenografts compared with celecoxib or radiation treatment alone (Fig. 7G, H). Moreover, TUNEL analysis revealed a marked increase of apoptotic cells in xenografts treated with celecoxib and radiation (Fig. 7I). Collectively, these results confirmed the concept that combined use of celecoxib sensitizes brain tumor to radiation.

## Discussion

Glioblastoma is one of the most refractory solid tumors because of its high heterogeneity [30]. Recent studies have consistently demonstrated that the high stemness and differentiative potential of GSCs lead to high heterogeneity, as well as tumor growth, immune escape, angiogenesis, and therapeutic resistance [31, 32]. Radiation and TMZ therapies induce serious DNA damage, including DNA double-strand break (DSB). Compared with tumor cells, GSCs possess mighty DNA damage repair ability [7, 33]. Our previous results showed that GSCs are more resistant to TMZ treatment than GBM cells. Moreover, hypoxic GSCs-derived EVs confer chemoresistance to GBM cells by delivering miR-30b-3p. Circulating miR-30b-3p is a potential biomarker for GBM patients [6]. A lot of lncRNAs have been proven to participate in GSCs growth and resistance to chemotherapy [34–36]. However, whether lncRNAs are involved in radiation therapy resistance remains unclear. In this study, we found a less studied lncRNA, LINC00839, was upregulated in GSCs, facilitating stemness maintenance and radiation resistance.

Recently, the biological significance of m6A modifications has drawn much attention for its vigoroso regulation on mRNAs and noncoding RNAs [37]. m6A modification is controlled by three types of regulators: writers, readers, and erasers [37]. Writers refer to the methyltransferases, which consist of METTL3, METTL14, WTAP, VIRMA, RBM15, and ZC3H13 [38]. METTL3 functions as an oncogene in GBM by modulating nonsense-mediated mRNA decay (NMD) of splicing factors and alternative splicing isoform switches [39]. K Somasundaram et al. demonstrated that SOX2 is a bona fide m6A target. Upon radiation treatment, Human antigen R (HuR) was recruited to SOX2 mRNA contributing to SOX2 stability mediated by METTL3 [40]. These results indicated that METTL3 is a potential molecular target for GBM therapy. In this study, we identified LINC00839 as an actual target of METTL3. METTL3 was overexpressed in GSCs and recurrent GBM samples, indicating METTL3 is a molecular regulator of GBM progression and recurrence.

“Readers” can recognize and bind m6A sites leading to the degradation or stabilization of target RNAs. Using RNA pull-down assays, we found LINC00839 could directly interact with YTHDF2. YTHDF2 initiates the decay and deadenylation of m6A mRNAs by recruiting the CCR4-NOT deadenylase complex [41]. YTHDF2 plays controversial roles in different kinds of tumor. In endometrioid endometrial carcinoma (EEC) cells, YTHDF2 mediates FENDRR degradation promoting tumor cell proliferation [42]. In glioma, YTHDF2 is essential for tumor cell proliferation by facilitating LXRA and HIVEP2 decay [43]. In GSCs, our group previously reported the proposed role of YTHDF2 in certain mRNAs stabilization and decay [18]. Consistent with previous results, in this study, we found that YTHDF2 was overexpressed in GSCs and recurrent GBM samples. Moreover, we observed that YTHDF2 specifically stabilizes LINC00839 in GSCs. Collectively, we further confirmed that YTHDF2 may be a therapeutic target of GBM.

Further mechanism research found that LINC00839 knockdown significantly suppressed Wnt/β-catenin signaling activation. Wnt/β-catenin signaling, one of the most evolutionarily conserved signaling, has been uncovered to play crucial roles in tumors [44]. In glioma, the aberrant activation of the Wnt/β-catenin pathway was observed [45]. A lot of molecular reasons have been proposed. For example, APC has been identified as a suppressor of Wnt/β-catenin signaling. A cohort analysis results indicated a 14.5% mutation frequency of APC in GBM patients, inducing Wnt/β-catenin signaling activation [46]. At the same time, dysregulation of lncRNAs were associated with Wnt/β-catenin signaling activation in GSCs. For instance, lncRNA MIR22HG is the host gene of miR-22-3p and miR-22-5p. Jian Wang et. al found that MIR22HG/miR-22 axis was overexpressed in GSCs. Bioinformation analysis accompanied by molecular biology experiments results suggested that silencing MIR22HG inhibited Wnt/β-catenin signaling activation through miR-22-3p and -5p [47]. In this study, we identified LINCOO839 directly interacted with β-catenin and c-Src. β-catenin Y654 phosphorylation by c-Src kinase makes it negatively charged and clashed with pivotal aspartate residues in cadherin, releasing β-catenin from the cellular membrane and Wnt signaling activation. Recent studies reported that lncRNAs could act as modular scaffolds of proteins, regulating their functions [48]. Knocking down LINC00839 significantly inhibited the interaction between c-Src and β-catenin, as well as β-catenin Y654 phosphorylation level, indicating that LINC00839 tied c-Src and β-catenin together, promoting β-catenin phosphorylation.

Celecoxib and other Wnt/β-catenin signaling inhibitors have been approved by FDA as effective anti-tumor drugs [49]. Celecoxib was originally designed as a COX-2 inhibitor to decrease NSAIDs-induced adverse reactions, and is used for the treatment of inflammatory diseases like rheumatoid arthritis [50]. Recently, celecoxib has been ascertained as a profound compound inhibiting Wnt/β-catenin signaling. Celecoxib treatment inhibited MGMT expression and Wnt/β-catenin signaling activation, thereby preventing chemoresistance [29]. Consistently, our results suggested that an additive or synergistic effect of celecoxib on GSCs in combination with radiation was observed. Celecoxib has been applied in several clinical trials. Sidney Kimmel Comprehensive Cancer Center at Johns Hopkins has initiated a clinical trail: Celecoxib in Patients With Newly Diagnosed GBM Who Are Receiving Anticonvulsant Drugs and Undergoing RT. The results confirmed that CPT-11 plus celecoxib could be safely administered, and this dosage regimen exhibits encouraging outcomes in recurrent GBM patients [51]. However, whether this response was generated by the inhibitory activity of celecoxib has not been well elucidated.

In conclusion, we elucidated the functions of LINC00839 in GSCs, and proposed the molecular mechanism model in which METTL3/YTHDF2 maintains LINC00839 expression in an m6A-dependent way. Stabilized LINC00839 functions as a scaffold guiding the binding and phosphorylation of c-Src on β-catenin, thereby promoting Wnt signaling activation. Combinational use of Wnt/β-catenin signaling inhibitor, celecoxib, sensitized brain tumors to radiation therapy in animal studies (Supplementary Fig. 8).

## Supplementary information

supplementary tables

supplementary figure legends

Figure S1

Figure S2

Figure S3

Figure S4

Figure S5

Figure S6

Figure S7

Figure S8

## Data Availability

The data used during the current study are available from the corresponding author on reasonable request.
