# Supplementary material for: METTL3-mediated m6A modification of LINC00839 maintains glioma stem cells and radiation resistance by activating Wnt/β-catenin signaling
Source: Cell Death Dis. 2023 Jul 12;14(7):417. doi: 10.1038/s41419-023-05933-7 (PMC10338500; doi:10.1038/s41419-023-05933-7)

**Table S1: Summary of clinical GBM patients**

| **Characteristic** | **All patient** | |  |
| --- | --- | --- | --- |
|  | **Primary**  **(n=30)** | **Recurrent**  **(n=20)** |  |
| **Sex (n)** |  |  |  |
| Male | 12 | 8 |  |
| Female | 18 | 12 |  |
| **age** |  | |  |
| ≥45 | 24 | 15 |  |
| ＜45 | 6 | 5 |  |
| **Tumor location** |  |  |  |
| Frontal | 11 | 9 |  |
| Non-frontal | 19 | 11 |  |
| **KPS score** |  | |  |
| ≥80 | 26 | 17 |  |
| ＜80 | 4 | 3 |  |
| **MGMT promotor status** |  |  |  |
| Methylated | 16 | 6 |  |
| Unmethylated | 14 | 14 |  |
| **Extent of surgery** |  | |  |
| Total | 21 | 10 |  |
| Subtotal | 19 | 10 |  |
| **IDH1/2 genotype** |  |  |  |
| Mutation | 4 | 2 |  |
| Wild-type | 26 | 18 |  |
|  |  |  |  |

**Abbreviations**: KPS, Karnofsky performance status; MGMT, O-6-methylguanine-DNA-methyltransferase; IDH1/2, isocitrate dehydrogenase 1 and 2.

| **Table S2 Primers used in this study** | | | |
| --- | --- | --- | --- |
| PCR |  |  |  |
|  | Forward | Reverse |  |
| LINC00839 | GACTTTGGAGCTGCCTCATGCA | ATCCAGGGAGAGTGCAGGCAAA | for qRT-PCR |
| GAPDH | GTCTCCTCTGACTTCAACAGCG | ACCACCCTGTTGCTGTAGCCAA | for qRT-PCR |
| Walking primers |  |  |  |
| 1 | ACTCTCCCTGGATGCTGG | TAGAGGTGTCAAGTGGGAAA |  |
| 2 | TTTTGCCTGCACTCTCCC | TAGAGGTGTCAAGTGGGAAA |  |
| 3 | CACTACTGGGTTGGGGTC | TAGAGGTGTCAAGTGGGAAA |  |
| 4 | GCCAAAACCAGCAACTGA | TAGAGGTGTCAAGTGGGAAA |  |
| 5 | TCTGCAGATGTGGATCCC | TAGAGGTGTCAAGTGGGAAA |  |
| RACE |  |  |  |
| LINC00839 | TGTTTGTGGCTCTCAGCTCTCAAGGCTG | | 3' CDS primer |
| LINC00839 | CATGGTGGGCTACTTCTCGCAGGATTCG | | 5' CDS primer |

| **Table S3. Full list of LINC00839 associated proteins** | | | |
| --- | --- | --- | --- |
| **Peptide Hits** | **Mass (KDa)** | **Gene Name** | **Full Name** |
| 33 | 92.168 | β-catenin | Catenin beta 1 |
| 30 | 28.329 | SRSF1 | Serine/arginine-rich splicing factor 1 |
| 30 | 82.968 | SEC23A | Protein transport protein SEC23 |
| 26 | 38.449 | SAE1 | SUMO-activating enzyme subunit 1 |
| 25 | 90.069 | RRM1 | Ribonucleoside-diphosphate reductase large subunit |
| 22 | 17.718 | RPS18 | 40S ribosomal protein S18 |
| 22 | 16.445 | RPS16 | 40S ribosomal protein S16 |
| 21 | 17.222 | RPS13 | 40S ribosomal protein S13 |
| 21 | 21.863 | RPL9 | 60S ribosomal protein L9 |
| 21 | 17.256 | RPL26L1 | 60S ribosomal protein L26-like 1 |
| 21 | 17.695 | RPL23A | 60S ribosomal protein L23a |
| 20 | 19.586 | RPL17 | 60S ribosomal protein L17 (Fragment) |
| 20 | 24.831 | RPL10A | 60S ribosomal protein L10a |
| 20 | 40.313 | RBM4 | RNA-binding protein 4 |
| 20 | 58.656 | RBM39 | Isoform 2 of RNA-binding protein 39 |
| 18 | 46.895 | RBM22 | Pre-mRNA-splicing factor RBM22 |
| 18 | 46.938 | RBBP7 | Histone-binding protein RBBP7 |
| 18 | 26.224 | RAN | GTP-binding nuclear protein Ran |
| 18 | 60.366 | c-Src | non-receptor tyrosine kinase |
| 17 | 28.48 | PSMB5 | Proteasome subunit beta type-5 |
| 17 | 28.147 | PSMA6 | Proteasome subunit alpha type |
| 16 | 40.422 | PSAT1 | Phosphoserine aminotransferase |
| 16 | 55.455 | PRPF31 | U4/U6 small nuclear ribonucleoprotein Prp31 |
| 16 | 25.035 | PRDX6 | Peroxiredoxin-6 |
| 16 | 32.118 | PNP | Purine nucleoside phosphorylase |
| 15 | 29.723 | PHB2 | Prohibitin |
| 15 | 85.018 | PFKL | ATP-dependent 6-phosphofructokinase, liver type |
| 13 | 37.497 | PCBP1 | Poly(rC)-binding protein 1 |
| 13 | 29.464 | NPM1 | Isoform 2 of Nucleophosmin |
| 13 | 32.642 | NME1-NME2 | Nucleoside diphosphate kinase |
| 13 | 79.372 | MTA1 | Metastasis-associated protein MTA1 |
| 12 | 46.513 | LUC7L2 | Putative RNA-binding protein Luc7-like 2 |
| 12 | 44.348 | HSP90AB2P | Putative heat shock protein HSP 90-beta 2 |
| 12 | 32.834 | HNRNPD | Isoform 3 of Heterogeneous nuclear ribonucleoprotein D0 |
| 12 | 55.102 | HDAC1 | Histone deacetylase 1 |
| 11 | 91.981 | GTF3C4 | General transcription factor 3C polypeptide 4 |
| 11 | 96.215 | GANAB | Neutral alpha-glucosidase AB |
| 11 | 54.529 | FSCN1 | Fascin |
| 11 | 100.83 | EXOSC10 | Exosome component 10 |
| 10 | 57.994 | EIF2A | Isoform 4 of Eukaryotic translation initiation factor 2A |
| 10 | 98.594 | DDX54 | ATP-dependent RNA helicase DDX54 |
| 9 | 28.299 | DDX39B | Spliceosome RNA helicase DDX39B (Fragment) |
| 9 | 86.233 | CUL3 | Isoform 2 of Cullin-3 |
| 9 | 31.703 | BUB3 | Mitotic checkpoint protein BUB3 (Fragment) |
| 9 | 104.55 | AP2B1 | AP-2 complex subunit beta |
| 9 | 34.352 | AIMP1 | Aminoacyl tRNA synthase complex-interacting multifunctional protein 1 |
| 9 | 66.514 | AIFM1 | Apoptosis-inducing factor 1, mitochondrial |
| 9 | 27.764 | YWHAQ | 14-3-3 protein theta |
| 9 | 108.58 | XRN2 | 5'-3' exoribonuclease 2 |
| 9 | 35.079 | WDR82 | WD repeat-containing protein 82 |
| 9 | 36.724 | WDR77 | Methylosome protein 50 |
| 9 | 66.193 | WDR1 | WD repeat-containing protein 1 |
| 9 | 49.775 | TUBB8 | Tubulin beta-8 chain |
| 9 | 54.708 | STAU1 | Double-stranded RNA-binding protein Staufen homolog 1 |
| 9 | 145.83 | SF3B1 | Splicing factor 3B subunit 1 |
| 8 | 22.127 | RPS7 | 40S ribosomal protein S7 |
| 8 | 18.079 | RPL18A | 60S ribosomal protein L18a |
| 8 | 46.158 | RBBP4 | Isoform 3 of Histone-binding protein RBBP4 |
| 8 | 52.904 | PSMD12 | 26S proteasome non-ATPase regulatory subunit 12 |
| 8 | 29.483 | PSMA4 | Proteasome subunit alpha type-4 |
| 8 | 68.283 | PGM2 | Phosphoglucomutase-2 |
| 8 | 70.182 | NXF1 | Nuclear RNA export factor 1 |
| 8 | 101.89 | MCM2 | DNA replication licensing factor MCM2 |
| 8 | 64.953 | MAGED2 | Melanoma-associated antigen D2 |
| 8 | 43.614 | LYAR | Cell growth-regulating nucleolar protein |
| 7 | 81.744 | JUP | Junction plakoglobin |
| 7 | 63.704 | IGF2BP3 | Insulin-like growth factor 2 mRNA-binding protein 3 |
| 7 | 86.371 | HADHA | Enoyl-CoA hydratase |
| 7 | 67.285 | GPI | Glucose-6-phosphate isomerase |
| 7 | 65.225 | GATAD2A | Isoform 2 of Transcriptional repressor p66-alpha |
| 7 | 61.64 | FUBP3 | Far upstream element-binding protein 3 |
| 7 | 273.2 | FASN | 3-hydroxyacyl-[acyl-carrier-protein] dehydratase |
| 7 | 42.502 | EIF3M | Eukaryotic translation initiation factor 3 subunit M |
| 6 | 41.581 | EIF3H | Eukaryotic translation initiation factor 3 subunit H |
| 6 | 45.745 | DNAJA2 | DnaJ homolog subfamily A member 2 |
| 6 | 50.519 | DDX19A | ATP-dependent RNA helicase DDX19A |
| 6 | 44.418 | CSNK2A1 | Casein kinase II subunit alpha |
| 6 | 97.621 | COPG2 | Coatomer subunit gamma-2 |
| 6 | 67.567 | CANX | Calnexin |
| 6 | 29.246 | CA2 | Carbonic anhydrase 2 |
| 5 | 48.043 | BZW1 | Basic leucine zipper and W2 domain-containing protein 1 |
| 5 | 57.56 | API5 | Apoptosis inhibitor 5 |
| 5 | 49.526 | AP2M1 | AP-2 complex subunit mu |
| 4 | 44.76 | ACTR2 | Actin-related protein 2 |
| 4 | 46.108 | ACAT1 | Acetyl-CoA acetyltransferase, mitochondrial |
| 4 | 45.476 | VRK1 | Serine/threonine-protein kinase VRK1 |
| 4 | 30.772 | VDAC1 | Voltage-dependent anion-selective channel protein 1 |
| 4 | 113.8 | UBA1 | Isoform 2 of Ubiquitin-like modifier-activating enzyme 1 |
| 4 | 53.12 | U2AF2 | Isoform 2 of Splicing factor U2AF 65 kDa subunit |
| 4 | 74.267 | TRAP1 | Isoform 2 of Heat shock protein 75 kDa, mitochondrial |
| 4 | 174.38 | TOP2A | DNA topoisomerase 2-alpha |
| 4 | 75.491 | TMPO | Lamina-associated polypeptide 2, isoform alpha |
| 4 | 76.979 | TBL3 | Transducin beta-like protein 3 (Fragment) |
| 4 | 25.542 | SRSF9 | Serine/arginine-rich splicing factor 9 |
| 4 | 61.494 | SNW1 | SNW domain-containing protein 1 |
| 4 | 22.391 | RPS5 | 40S ribosomal protein S5 (Fragment) |
| 4 | 14.839 | RPS15A | 40S ribosomal protein S15a |
|  |  |  |  |
| Note: Those proteins with peptide hits ≥ 4 are shown. | | | |

**TableS4**
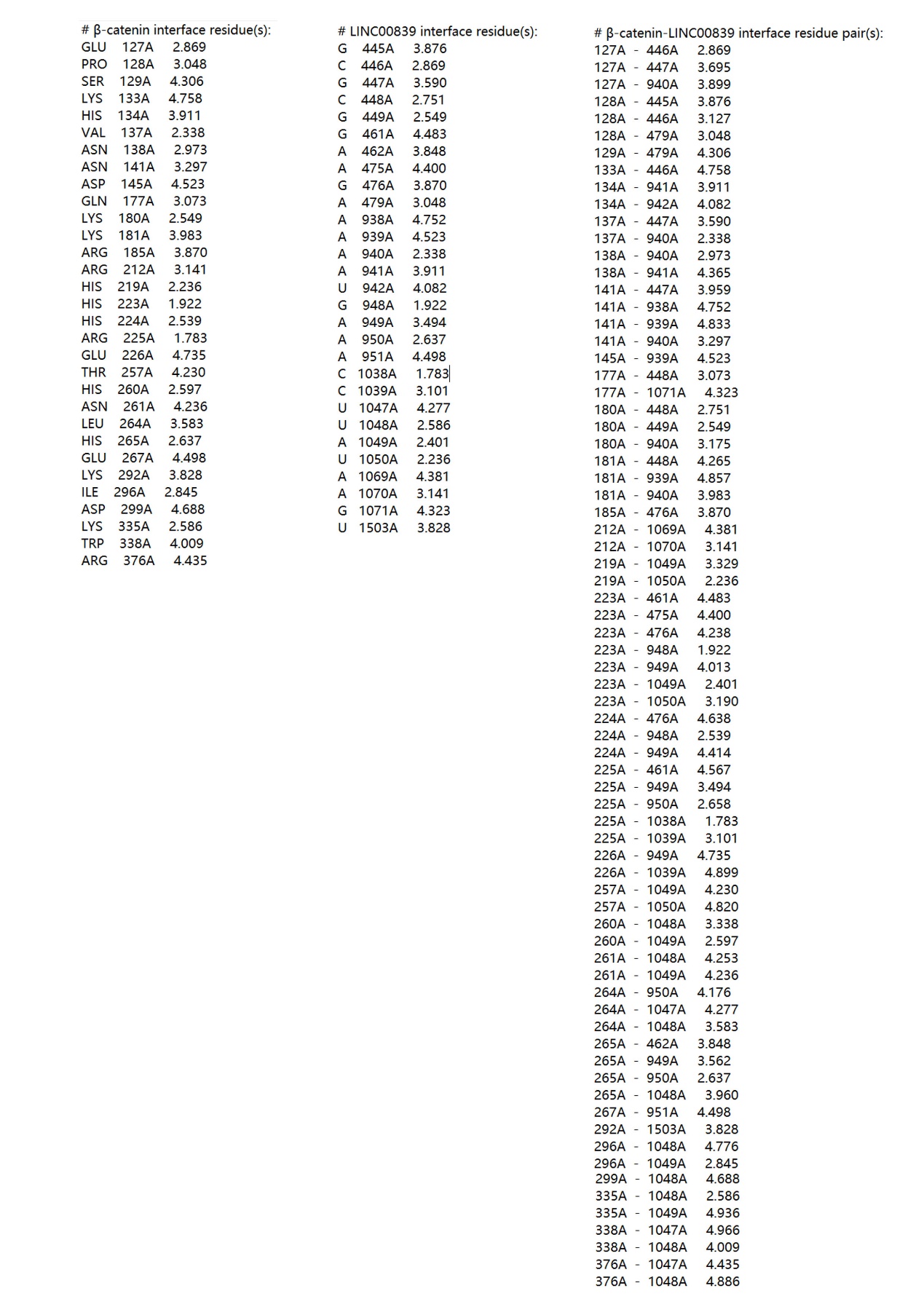

Supplement: Supplementary file 1 — supplementary tables [file 41419_2023_5933_MOESM1_ESM.docx]
