# Supplementary material for: METTL3-mediated m6A modification of LINC00839 maintains glioma stem cells and radiation resistance by activating Wnt/β-catenin signaling
Source: Cell Death Dis. 2023 Jul 12;14(7):417. doi: 10.1038/s41419-023-05933-7 (PMC10338500; doi:10.1038/s41419-023-05933-7)
Supplement: Supplementary file 2 — supplementary figure legends [file 41419_2023_5933_MOESM2_ESM.docx]

**Supplementary figure legends**

**Figure S1**

A. PAX8-AS1, MIR4458HG, LINC02732, and ELFN1-AS1 expression in NSCs and GSCs was detected by qRT-PCR.

B. Schematic representation of exons and transcripts of LINC00839 and its loci on human chromosome 10q11.21 in UCSC Genome browser.

C. The half-life of LINC00839 in MES28 cells treated with 2.5 μM actinomycin D. β-actin served as the internal control.

D. Representative images of PCR products from the 5’ RACE and 3’ RACE.

E. The sequence identified by RACE assays are underlined.

F. Coding potential of LINC00839 analyzed by LNCipedia.

G. *In vitro* transcription and translation of LINC00839 sense or antisense transcript. Luciferase (Luc) is used as a positive control.

H. The enrichment of LP0 in pull-down products of LINC00839 was measured by western blot.

**Figure S2**

A. Overexpression and knockdown of LINC00839 in GSCs were confirmed by qRT-PCR. ** *p* < 0.001.

B. LINC00839 was overexpressed in GSCs. Expression of stemness markers were detected by western blot.

C. LINC00839 was depleted in GSCs. Expression of stemness markers were detected by western blot.

D. MES28 cells were transfected with vector or LINC00839. The apoptotic rates were measured by flow cytometry. ** *p* < 0.001.

E and F. GSC2907 cells were transfected with LINC00839 ASO (E) or LINC00839 (F). The apoptotic rates were measured by flow cytometry. ** *p* < 0.001.

G. GSCs cells were transfected with LINC00839 (G) or LINC00839 ASO (H). The dynamic expression of γ-H2AX were detected.

**Figure S3**

A. METTL3 expression in NSCs and GSCs was detected by qPCR.

B. WTAP expression in NSCs and GSCs was detected by qPCR.

C. WTAP expression in NSCs and GSCs was detected by western blot.

D. METTL3 knockdown in GSCs were confirmed by western blot.

E. METTL3 overexpression in GSCs were confirmed by western blot.

F. GSCs were treated with control or METTL3 shRNA. LINC00839 expression was detected by qRT-PCR. ** *p* < 0.001.

G. Total RNA m6A contents in LINC00839-knockdown GSCs was quantified by m6A levels quantification analysis.

H. The enriched and specific m6A peak distribution of LINC00839 transcripts predicted by SRAMP.

I. The eight m6A modification sites in LINC00839 were predicted by SRAMP.

J. Schematic diagram of the WT and 6A-mutated plasmid.

**Figure S4**

A. The enrichment of “Readers” in pull-down products of LINC00839 was measured by western blot.

B. YTHDF2-LINC00839 interaction was predicted by RPISeq.

C. YTHDF2 expression in NSCs and GSCs was detected by western blot.

D. YTHDF2 expression in NSCs and GSCs was detected by qPCR. ** *p* < 0.001.

E. YTHDF2 knockdown in GSCs were confirmed by western blot.

F. YTHDF2 overexpression in GSCs were confirmed by western blot.

G. GSCs with or without expression of YTHDF2 shRNA were reconstituted with or without expression of WT Flag-YTHDF2 or Flag-YTHDF2 W432A or W486A protein.

**Figure S5**

A. FISH and IF double staining in GSCs showing the co-localization of LINC00839 (Cy3; Red) and β-catenin (Green); Nuclei are stained blue (DAPI). Scale bar: 10 μm.

B. The secondary structure of LINC00839 was analyzed by RNAfold. The section circled in red is 445-1503 nt fragment of LINC00839.

C. GSCs were transfected with FL or TL LINC00839. Effects on self-renewal were assessed by *in vitro* extreme limiting dilution assays (ELDA) for sphere formation. * refers to compare between Vector group and FL group; # refers to compare between FL group and TL group. ** *p* < 0.001, ## *p* < 0.001.

D. GSCs were transfected with FL or TL LINC00839. Expression of stemness markers were detected by western blot.

E. GSCs were transfected with FL or TL LINC00839. The apoptotic rates were measured by flow cytometry. ** *p* < 0.001.

F. GSCs were transfected with FL or TL LINC00839. The dynamic expression of γ-H2AX were detected.

**Figure S6**

A and B. GSCs were transfected with vector or LINC00839. β-catenin expression were measured by qPCR (A) and western blot (B).

C and D. GSCs were transfected with control or LINC00839 ASO. β-catenin expression were measured by qPCR (C) and western blot (D).

E. GSCs were fractionated into indicated cellular components after transfected with control or LINC00839 ASO. β-catenin expression were measured by western blot.

F. Luciferase activity (TOP/FOP) in GSCs transfected with control or LINC00839 ASO. ** *p* < 0.001.

G. Control or METTL3 deleted GSCs were co-transfected with LINC00839. Luciferase activity (TOP/FOP) was measured.

H. Control or YTHDF2 deleted GSCs were co-transfected with LINC00839. Luciferase activity (TOP/FOP) was measured.

I. GSCs were fractionated into indicated cellular components after transfected with FL or TL LINC00839. β-catenin expression were measured by western blot.

J. Luciferase activity (TOP/FOP) in GSCs transfected with FL or TL LINC00839. ** *p* < 0.001.

K: GSCs with or without expression of β-catenin shRNA were reconstituted with or without expression of WT Flag-β-catenin or Flag-β-catenin Y654E protein.

L. Luciferase activity (TOP/FOP) in control or LINC00839 overexpressing GSC2907 co-transfected with WT or Y654E mutation β-catenin. ** *p* < 0.001.

M. Control or LINC00839 overexpressing GSCs were co-transfected with WT or Y654E mutation β-catenin. Expression of stemness markers were detected by western blot.

N. Control or LINC00839 overexpressing GSCs were co-transfected with WT or Y654E mutation β-catenin. Cells were treated with or without IR (0–10 Gy) and seeded in 10 cm dishes. Colonies were counted after 2 weeks, and the surviving fraction was calculated as the ratio of the plating efficiency of the treated cells to that of control cells. ** *p* < 0.001.

**Figure S7**

A. Dose-response curves of Wnt/β-catenin inhibitors treatment in MES28. IC_50_ of Wnt/β-catenin inhibitors for MES28 was measured using nonlinear regression analysis of the dose-response curves.

B. GSCs were treated with increased dose of celecoxib. Luciferase activity (TOP/FOP) was measured. ** *p* < 0.001.

C. GSCs were treated with increased dose of celecoxib. Expression of stemness markers were detected by western blot.

D. GSCs were treated with increased dose of celecoxib. The apoptotic rates were measured by flow cytometry. ** *p* < 0.001.

E. GSCs were treated with increased dose of celecoxib. The dynamic expression of γ-H2AX were detected.

**Figure S8 Working model of this study**
