# Supplementary figures and images for: METTL3-mediated m6A modification of LINC00839 maintains glioma stem cells and radiation resistance by activating Wnt/β-catenin signaling
Source: Cell Death Dis. 2023 Jul 12;14(7):417. doi: 10.1038/s41419-023-05933-7 (PMC10338500; doi:10.1038/s41419-023-05933-7)

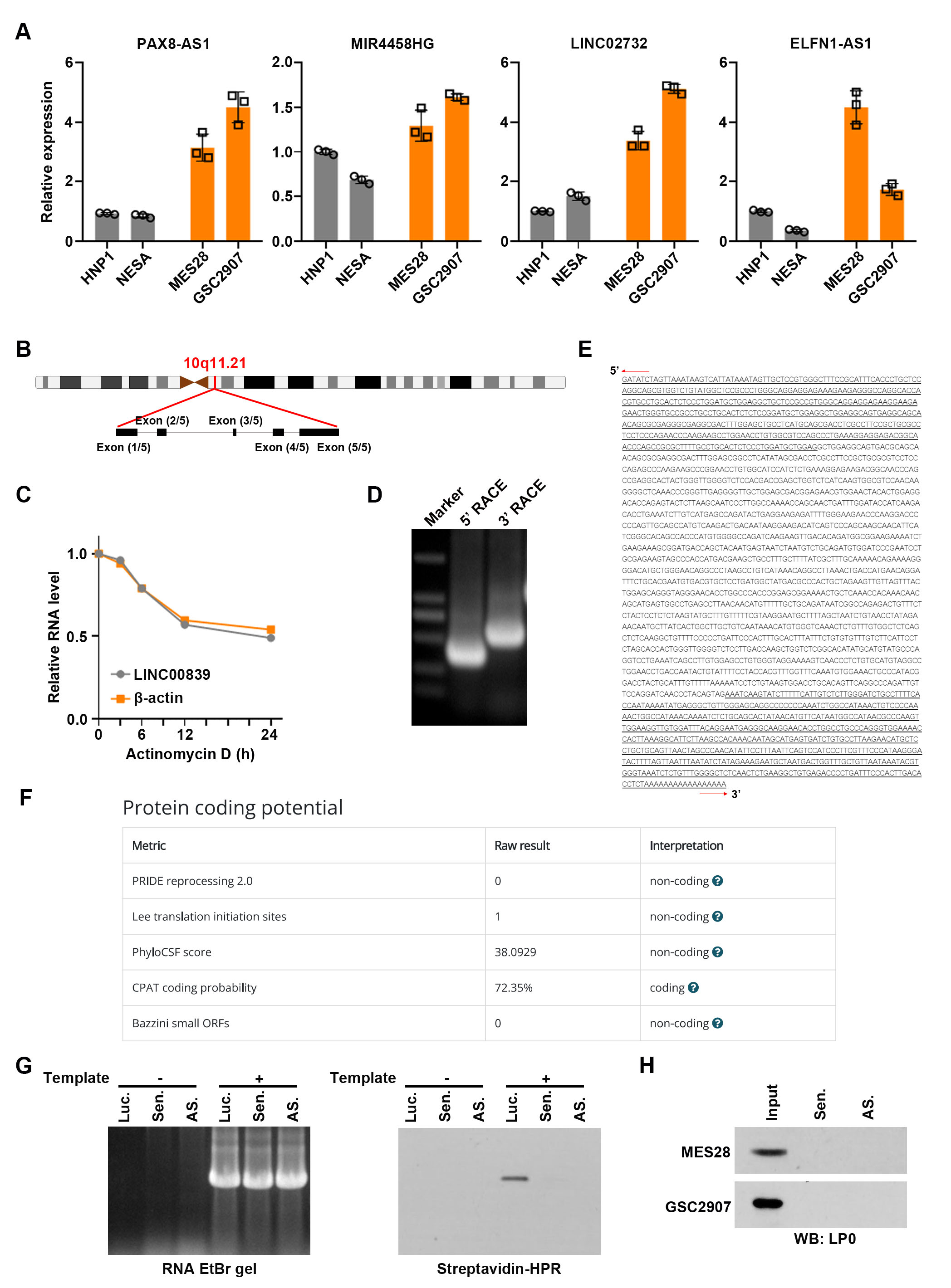

Supplement: Supplementary file 3 — Figure S1 [file 41419_2023_5933_MOESM3_ESM.jpg]

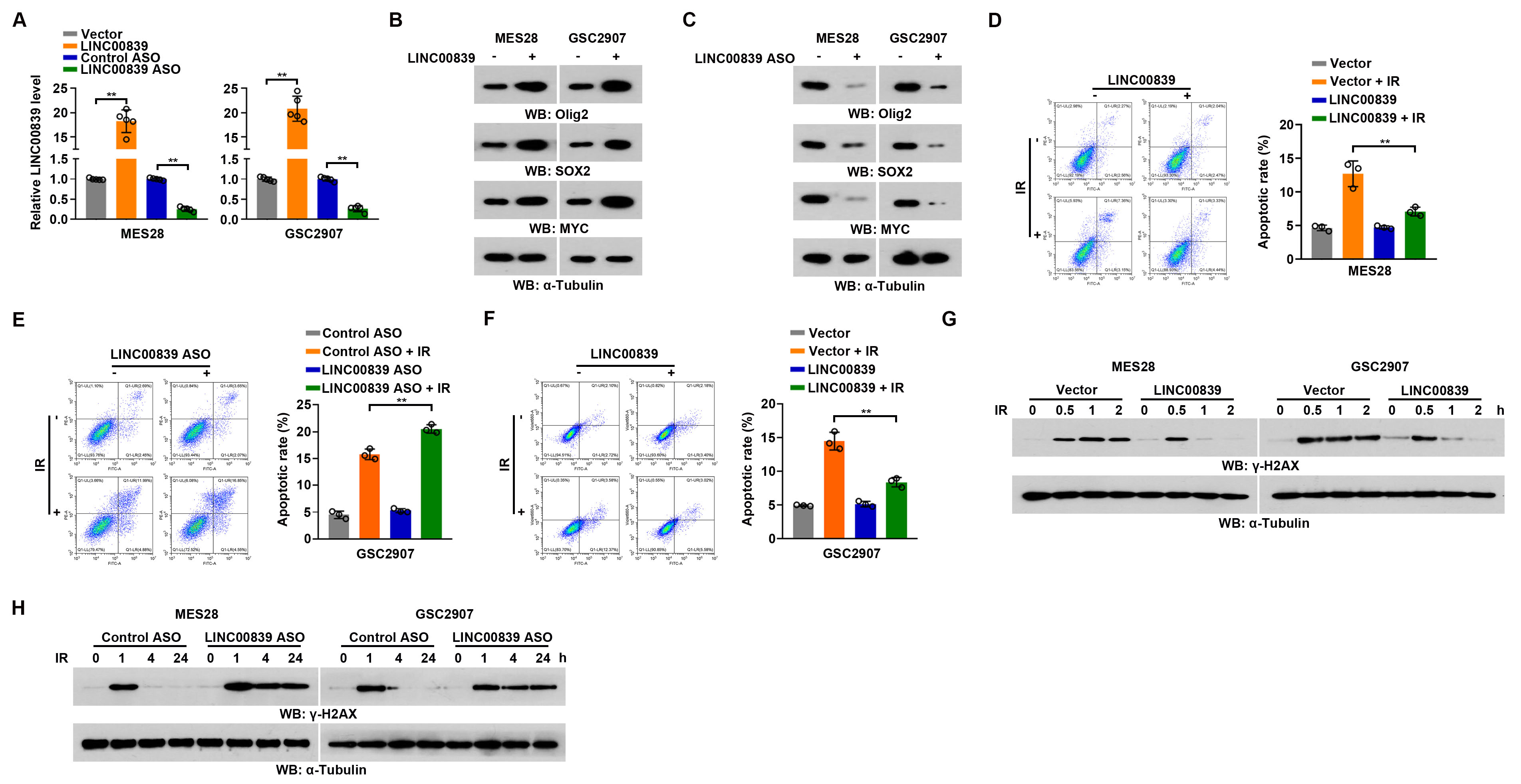

Supplement: Supplementary file 4 — Figure S2 [file 41419_2023_5933_MOESM4_ESM.jpg]

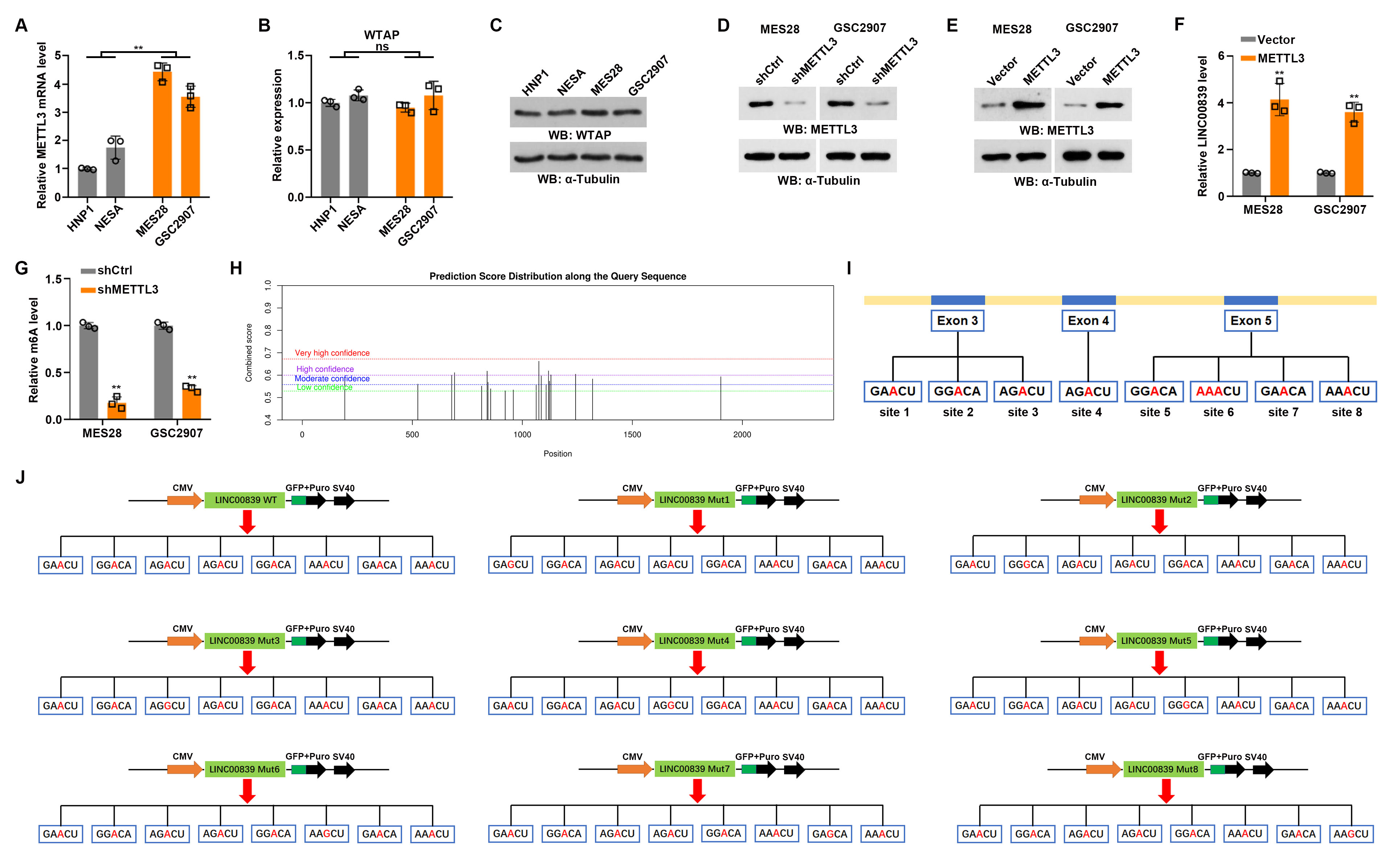

Supplement: Supplementary file 5 — Figure S3 [file 41419_2023_5933_MOESM5_ESM.jpg]

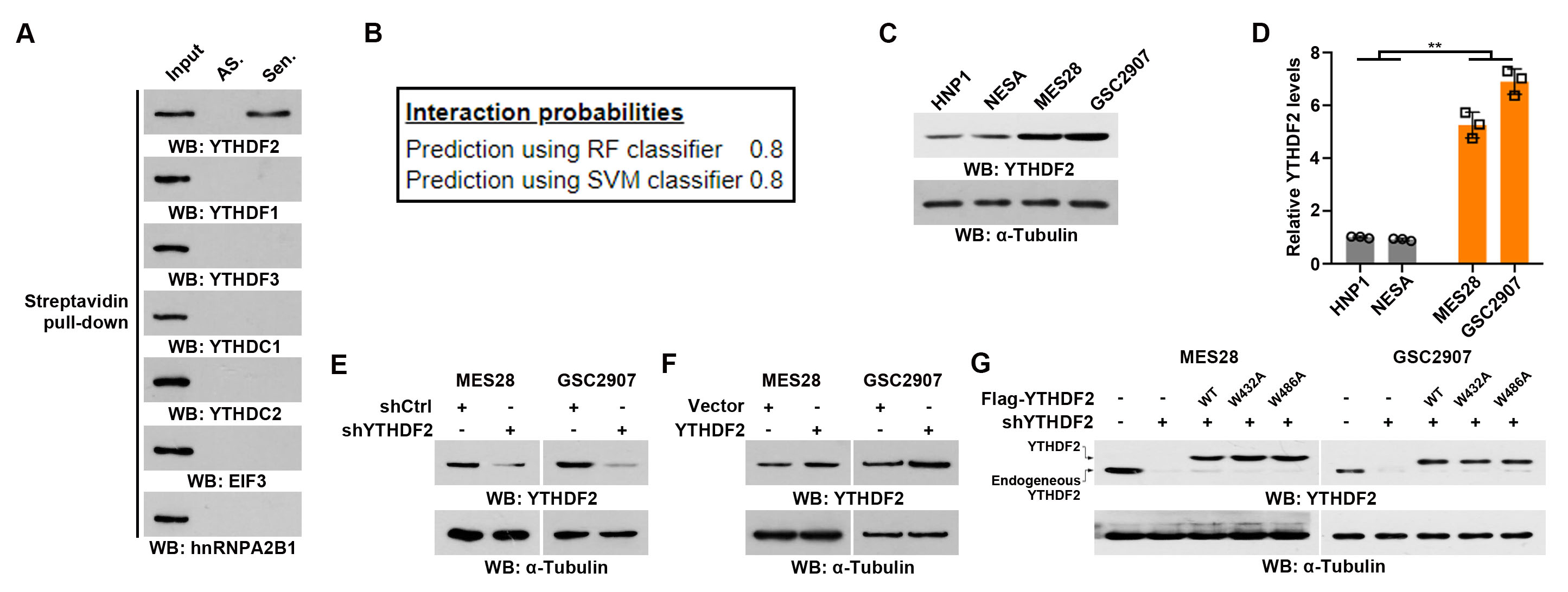

Supplement: Supplementary file 6 — Figure S4 [file 41419_2023_5933_MOESM6_ESM.jpg]

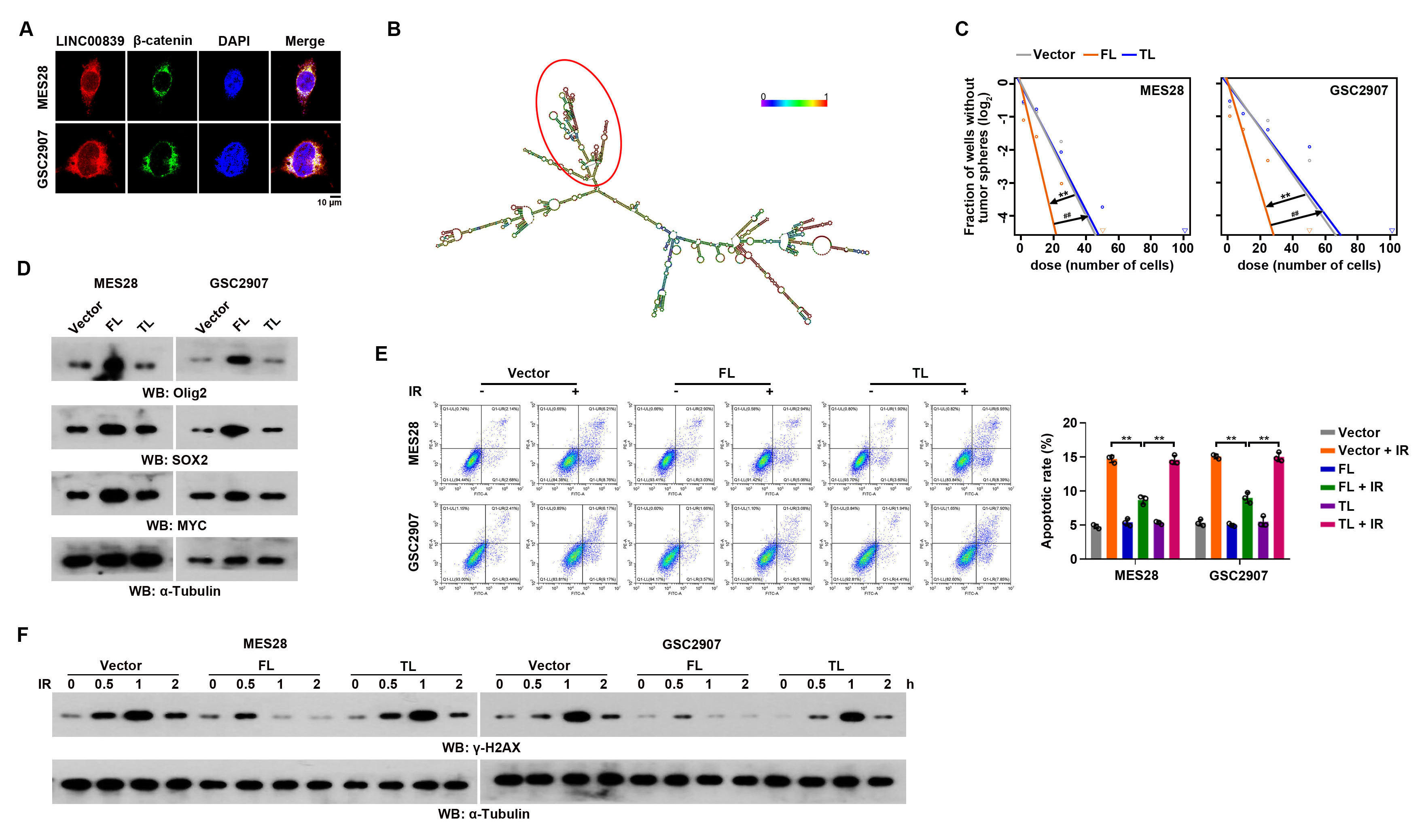

Supplement: Supplementary file 7 — Figure S5 [file 41419_2023_5933_MOESM7_ESM.jpg]

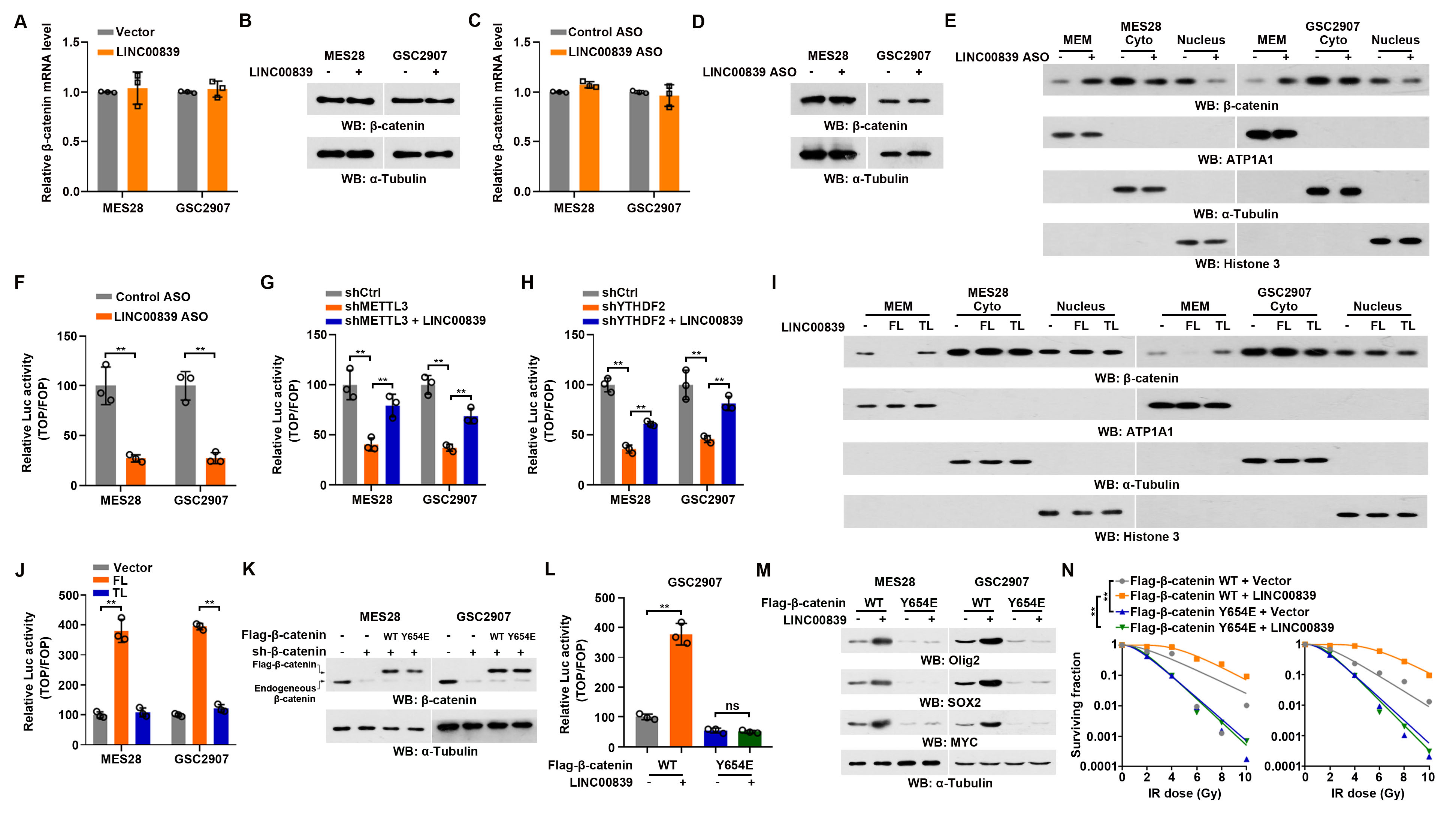

Supplement: Supplementary file 8 — Figure S6 [file 41419_2023_5933_MOESM8_ESM.jpg]

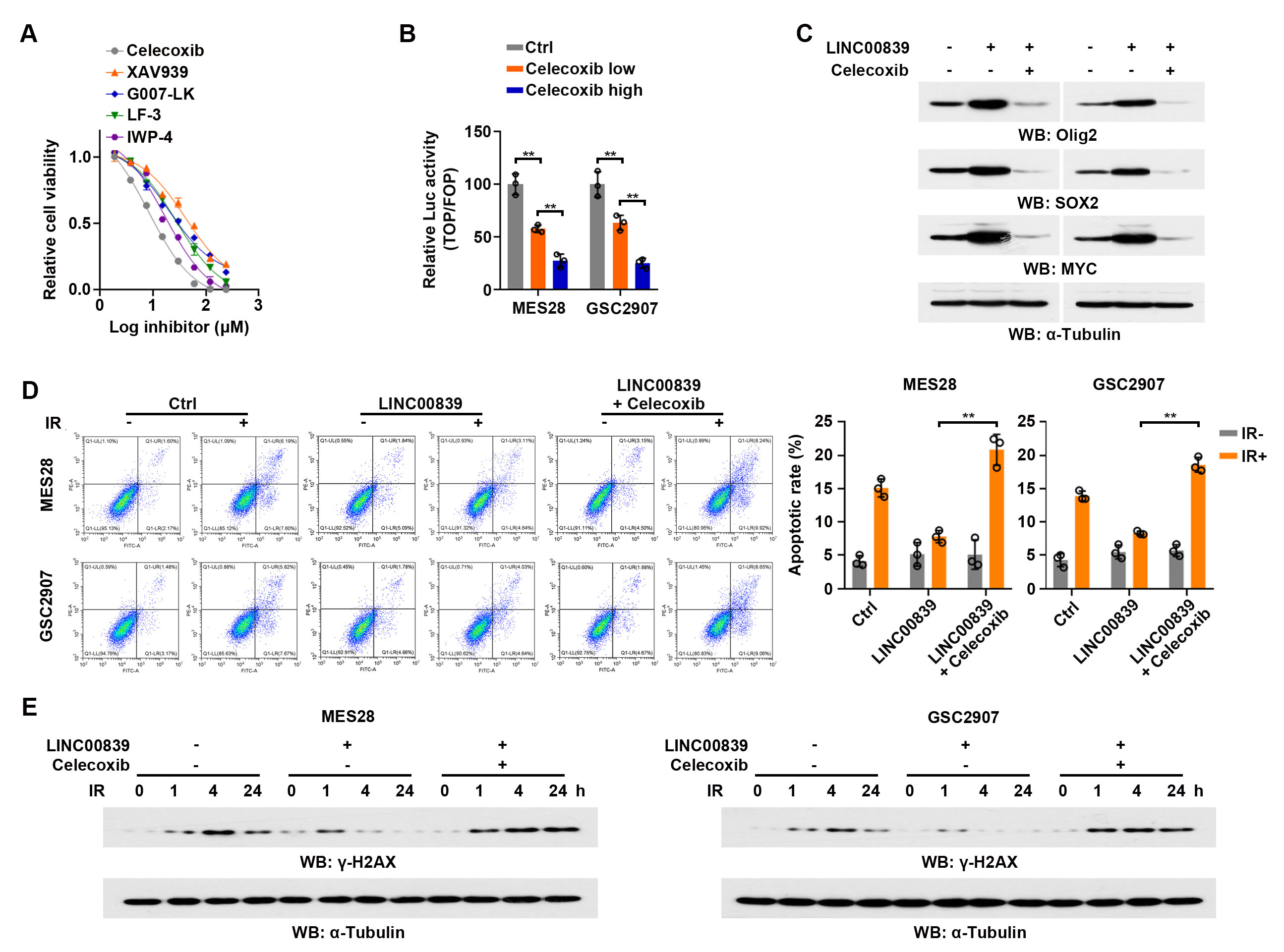

Supplement: Supplementary file 9 — Figure S7 [file 41419_2023_5933_MOESM9_ESM.jpg]

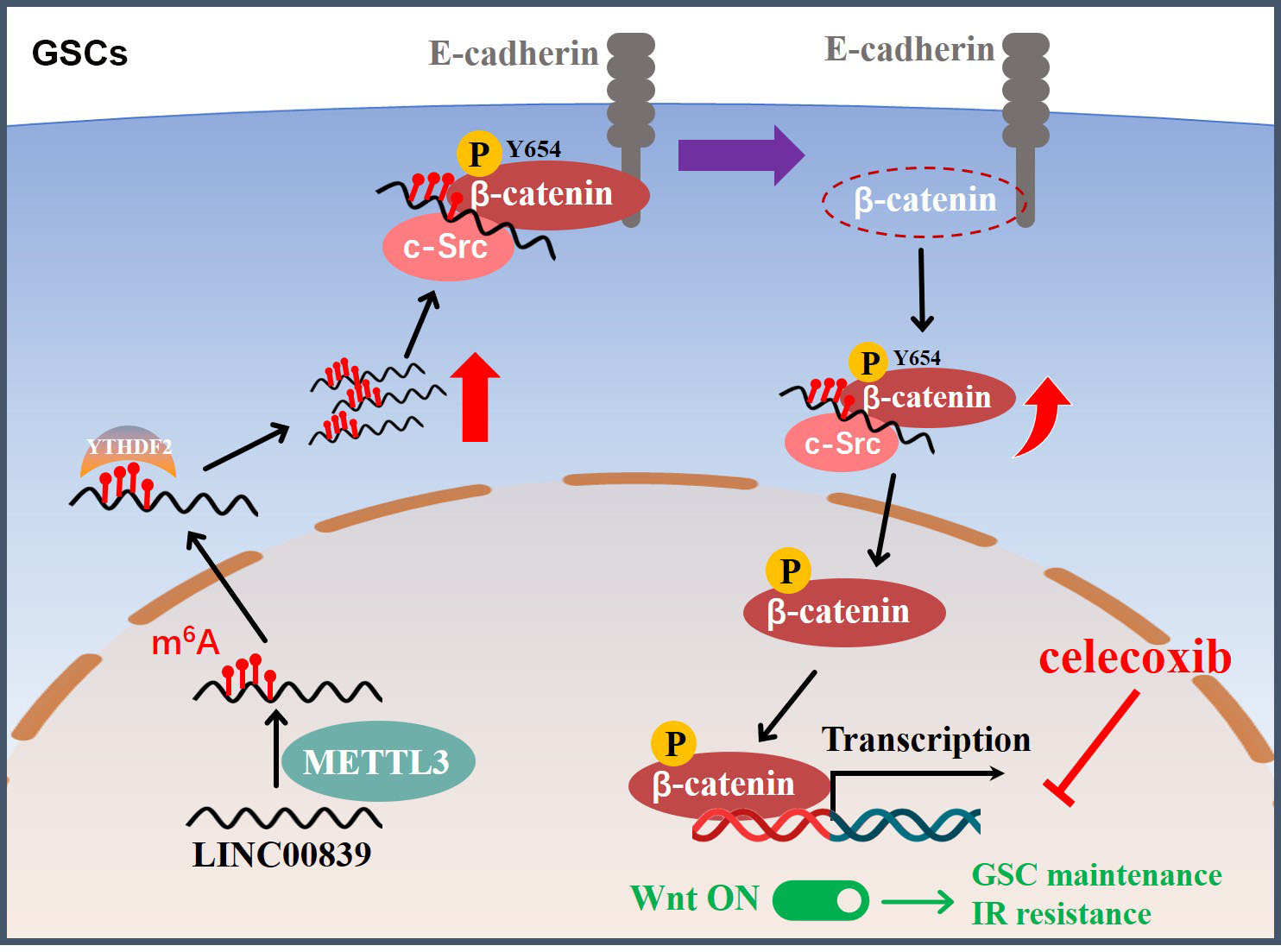

Supplement: Supplementary file 10 — Figure S8 [file 41419_2023_5933_MOESM10_ESM.jpg]
